# Distribution of deep-water corals, sponges, and demersal fisheries landings in Southern California, USA: implications for conservation priorities

**DOI:** 10.7717/peerj.5697

**Published:** 2018-10-10

**Authors:** Enrique J. Salgado, Stephanie E. Nehasil, Peter J. Etnoyer

**Affiliations:** 1 NOAA National Centers for Coastal Ocean Science, Charleston, SC, USA; 2 CSS Inc., Fairfax, VA, USA; 3 Division of Biological Sciences, Ecology, Behavior, and Evolution Section, University of California, San Diego, La Jolla, CA, USA

**Keywords:** Deep-sea, Corals, Bottom fishing, ROV, California, Fisheries, Marine protected areas, Bycatch, Habitat, Marine debris

## Abstract

Deep-sea corals in Southern California are diverse and abundant but subject to multiple stressors, including bottom-contact fisheries using mobile and fixed gear. There is a need for more information on the distribution of these taxa in relation to the distribution of demersal fishing effort, and the distribution of marine protected areas, in order to improve spatial planning. There are many marine managed areas in Southern California, including essential fish habitat (EFH) areas, conservation areas, and a national marine sanctuary, but specific areas of overlap between bottom fishing and benthic epifauna are poorly known. Groundfish surveys were conducted by the National Marine Fisheries Service using a remotely operated vehicle throughout Southern California between 2003 and 2011 to document abundance and distribution of deep-water rockfish and flatfish to a depth of 500 m. Corals and sponges were also common in these images, providing an opportunity to examine these communities. Analyses of 34,792 still images revealed abundance and diversity of coral and sponge taxa, as well as frequency of fishing debris. The occurrence data were overlaid in a geographic information system with landings data for deep-water (>50 m) demersal fisheries to identify areas of spatial overlap. Corals or sponges were observed in 23% of images. A total of 15 coral genera and six sponge morphotypes were identified. A total of 70 species codes were targeted by deep-water demersal fisheries operating below 50 m for years 2007–2011. A novel priority-setting algorithm was developed to identify areas of high richness, abundance, and fishing intensity (RAFi). Several highly-ranked areas were already protected as EFH (Footprint, Piggy Bank). Other highly-ranked sites (West Catalina Island, San Clemente Island, 9-Mile Bank, Santa Rosa Flats) were encompassed by transient gear restrictions, such as Rockfish conservation areas, but are now recommended for permanent protection by the Pacific Fishery Management Council.

## Introduction

Deep-water (>50 m) azooxanthellate coral and sponge communities are widespread in the Northeast Pacific, from the Bering Sea west to the Hawaiian Islands and south to the tip of Baja California (Verrill, 1870; Ostarello, 1973). In this region, deep-water coral species are reported from 50 to 3,880 m, but are most abundant between 50 and 1,000 m (Etnoyer & Morgan, 2005). These deep-water communities provide habitat for fishes and invertebrates (Husebø et al., 2002; Ross & Quattrini, 2007; Love et al., 2007; Stone, 2006) and they are considered important components of deep-sea benthic ecosystems that provide areas for juvenile development and shelter from predation (Baillon et al., 2012).

The complex bathymetry of the seafloor and cold, highly productive upwelling oceanography of the California Current Ecosystem are conducive to deep-water coral and sponge growth within the Southern California Bight (SCB; Point Conception, CA, to the U.S.—Mexico Border). Habitat suitability models based on topographic and environmental data and a growing National Database of Deep-Sea Coral occurrences (Hourigan et al., 2015) suggest high likelihood for several families of corals in the waters surrounding the islands of San Nicolas, Santa Catalina, and San Clemente (Huff et al., 2013; Guinotte & Davies, 2014).

Deep-water azooxanthellate corals have been documented in the SCB since the 19th century, particularly the colorful hydrocoral *Stylaster californicus* (Verrill, 1866; Ostarello, 1973), the reef-building scleractinian coral *Lophelia pertusa* (Durham, 1947; Cairns, 1994; Hardin et al., 1994) and the solitary black coral *Antipathes dendrochristos* (Opresko, 2005; Tissot et al., 2006; Love et al., 2007). A variety of previously described structure-forming sessile benthic invertebrates, including many species of sponges, sea pens, and gorgonians, have been identified throughout the SCB using human-occupied submersibles (Tissot et al., 2006), towed camera sleds (Krigsman et al., 2012), and autonomous underwater vehicles (Whitmire & Clarke, 2007). Furthermore, several new, undescribed species of carnivorous sponges (Reiswig & Lee, 2007) and gorgonians (Etnoyer, 2008; Williams, 2013) were recently discovered using remotely operated vehicles (ROVs).

Due to the challenges and high costs of surveying remote deep-sea ecosystems, much remains unknown about the distribution and abundance of corals and sponges in these areas. Much of what is known about deep-sea corals and sponges in the SCB is based on museum records (Etnoyer & Morgan, 2005) and published observations from submersible expeditions (Tissot et al., 2006; Love et al., 2007; Yoklavich et al., 2011). Other records come from telepresence expeditions using ROVs and incidental bycatch in commercial fisheries. These records are compiled through NOAA’s National Database of Deep-Sea Corals (NOAA, 2017), but to date there has been no synthesis of these occurrences in relation to fisheries landings in the SCB, such as the analysis provided here.

The limited information that is available on these sessile organisms suggests that they are potentially vulnerable to disturbance from bottom-contact fishing gear. Deep-sea corals and sponges are slow-growing and often live hundreds of years (Roark et al., 2005; Andrews et al., 2002). These corals and sponges also co-occur with many commercially important fish species that also inhabit deep, rocky substrates, and therefore may be negatively impacted by bottom-contact fixed gear (e.g., set nets, longlines, and traps) and mobile gear (e.g., trawls) in these areas (Morgan et al., 2005; Whitmire & Clarke, 2007; Parker & Bowden, 2010; Shester & Micheli, 2011; Rooper et al., 2017). Closed areas have been established that provide corals and sponges with permanent (e.g., essential fish habitat (EFH), marine reserves) or temporary (e.g., fishery conservation areas) de facto protections from some bottom-contact fishing activities (California Department of Fish and Wildlife, 2016), but these temporary areas may be reopened to bottom fishing once target fish populations rebuild (Pacific Fishery Management Council, 2006).

Fishery management plans for groundfish describe EFH as the waters and substrate needed for breeding, feeding, and growth to maturity (Pacific Fishery Management Council, Amendment 19, 2006). EFH areas that are important to ecological function, rare, or sensitive to environmental degradation and development are identified as “habitat areas of particular concern” (HAPC). Estuaries, kelp beds, seagrass, and rocky reefs are types of HAPC for groundfish. Rocky reefs are considered the least abundant but most important HAPC (Pacific Fishery Management Council, Amendment 19, 2006) and are often invoked as the best habitat for corals and sponges (Tissot et al., 2006; Whitmire & Clarke, 2007; Yoklavich et al., 2011). There is a legal mandate in the Magnuson-Stevens Fishery Conservation and Management Act (2006) to minimize adverse impacts to EFH as well as discretionary authority in the Act to protect deep-sea coral ecosystems.

The objectives of this study were to examine the spatial relationships between deep-water bottom-contact fisheries and deep-sea corals and sponges in the SCB. We compared data on the abundance and diversity of deep-sea corals and sponges from ROV surveys with the frequency of observed derelict fishing gear and fishery landings (2007–2011) from adjacent areas (Perry et al., 2010; California Department of Fish and Wildlife, 2011). This was done in order to examine areas of potential overlap across the region and within particular depth ranges. The period from 2007 to 2011 is a temporal frame of reference for fisheries managers, since several protected areas were established in 2006. California Department of Fish and Wildlife (CDFW) catch block data has been used to track the distribution of fisheries along the California coast since 1935 (Miller et al., 2014).

Benthic images were used to estimate diversity and abundance of corals and sponges at the mesoscale of a feature, or named place, referred to as a site. Many ROV dives occurred at each site, and many images were taken on each dive. The images captured information on number, size, and identity of organisms and derelict fishing gear, as well as their frequency of occurrence. These data were combined with regional fishery landings to develop a novel conservation index used to identify areas where interactions between fisheries and sensitive habitats are likely to have occurred, and where there is high diversity and abundance, but little evidence of fishing. This study does not address indirect impacts from fishing; however, the RAFi index is consistent with the goals of HAPC to identify habitats that are sensitive or rare, in terms of abundance and diversity; or stressed, in terms of potential fishing impacts.

## Methods

### Survey area and image collection

**Figure 1:**
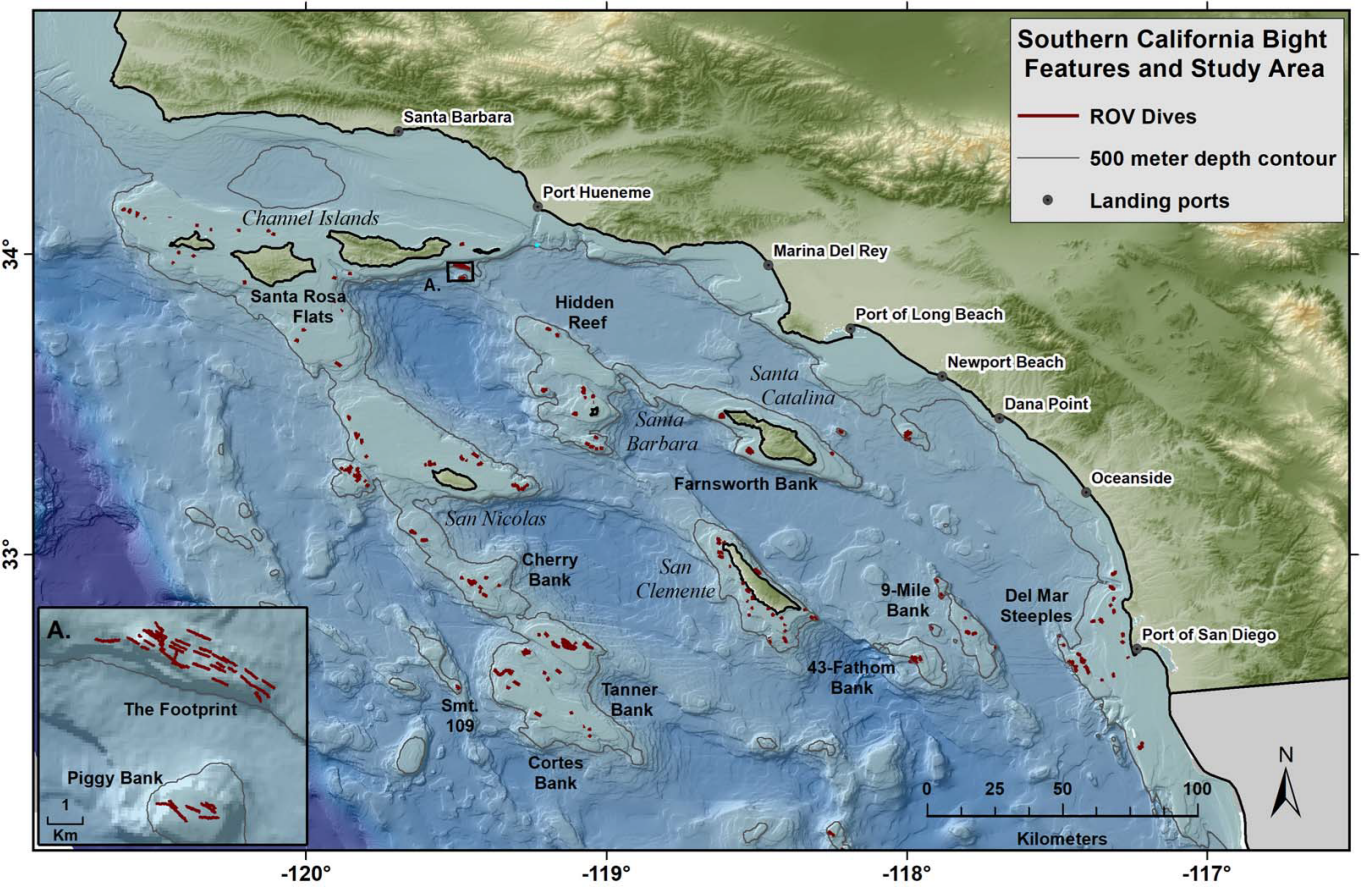
Map of study area, islands, topography, and ports. Red lines show the tracks of 352 ROV dives by NOAA within the Southern California Bight over the course of a 9 year study. The study was limited to a maximum depth of 500 m.

High-resolution still images were collected during 352 ROV transects conducted during surveys of abalone *Haliotis* sp. (Stierhoff, Neuman & Butler, 2012) and rockfishes *Sebastes* and *Sebastolobus* spp. (Demer, 2012) in SCB between the depths of 27 and 579 m during the period between November 5th, 2003 and December 7th, 2011 (Fig. 1). Transects were haphazard, focused on predominantly rocky areas with moderate relief, generally working from deep-water to shallow water over an average distance between one and two km per dive. During these surveys, still photographs were collected periodically to aid identification of demersal fishes and abalone; however, many corals and sponges were also documented. Still photographs of non-target corals and sponges were collected opportunistically. All unique images where the bottom was visible were used to describe the distribution of corals and sponges in the study area.

A total of 34,792 still images were collected at 32 banks and ridges throughout the SCB (Fig. 1). Photos were collected using a three megapixel digital still camera (Scorpio; Insite Pacific, Inc., Solana Beach, CA, USA.) attached to an ROV (Phantom DS4; Deep Ocean Engineering, Inc., San Jose, CA, USA). Two pairs of parallel lasers spaced at 20 and 61 cm apart provided a size reference. A conductivity, temperature, and depth (CTD) sensor (Citadel CTD; Teledyne RD Instruments, Inc., Poway, CA, USA) and oxygen optode (Model 3975; Aanderaa Data Instruments AS, Bergen, Norway) were used to describe the physical environment for each image. The location of the ROV above the seabed was determined using an ultra-short baseline acoustic tracking system (TrackPoint-II; ORE Offshore Technologies, Inc.) with a differential GPS (CSI Wireless, Inc. Calgary, AB Canada). All ROV data (e.g., latitude and longitude, physical data) and photographs were synchronously time-stamped and georeferenced.

**Figure 2:**
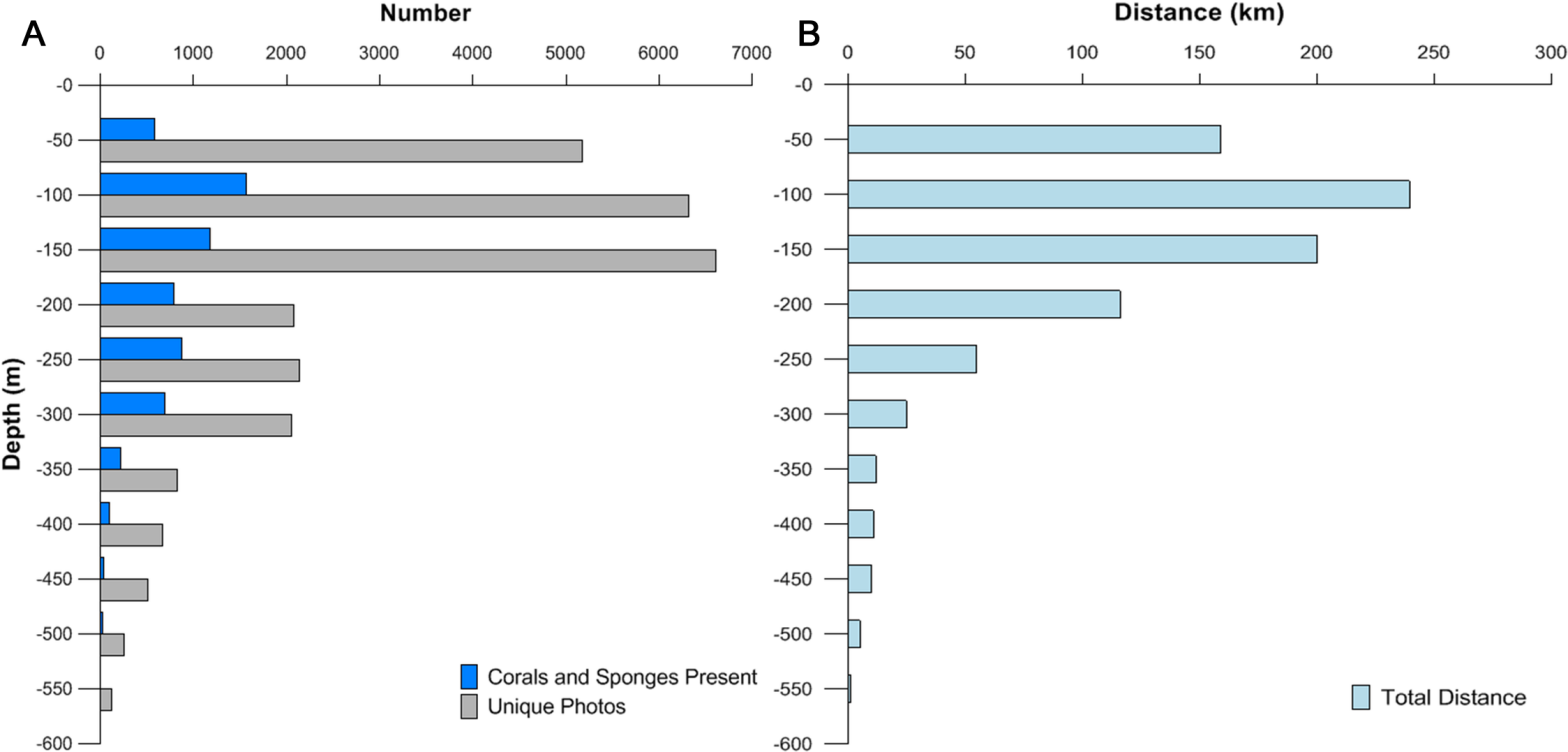
Depth distribution of images, corals and sponges, and survey effort. (A) The numbers of images collected (gray), and images containing corals, sponges, or both (blue). (B) Total survey effort (distance) for each 50 m depth category.

The majority (57.5%) of images were from mesophotic depth zones between 45 and 150 m (Fig. 2A). The lengths of transects varied depending on the survey type, feature size, and geological characteristics. Survey effort was quantified by summing the lengths of each ROV transect in each depth bin (Fig. 2B). An average of 7,500 images were collected from each of three 50 m interval bins <150 m. An average of 2,500 images were collected from bins between 151 and 350 m. The number of images available per depth class dropped below 1,000 in zones deeper than 351 m.

### Identification and classification of corals, sponges, and debris

**Figure 3:**
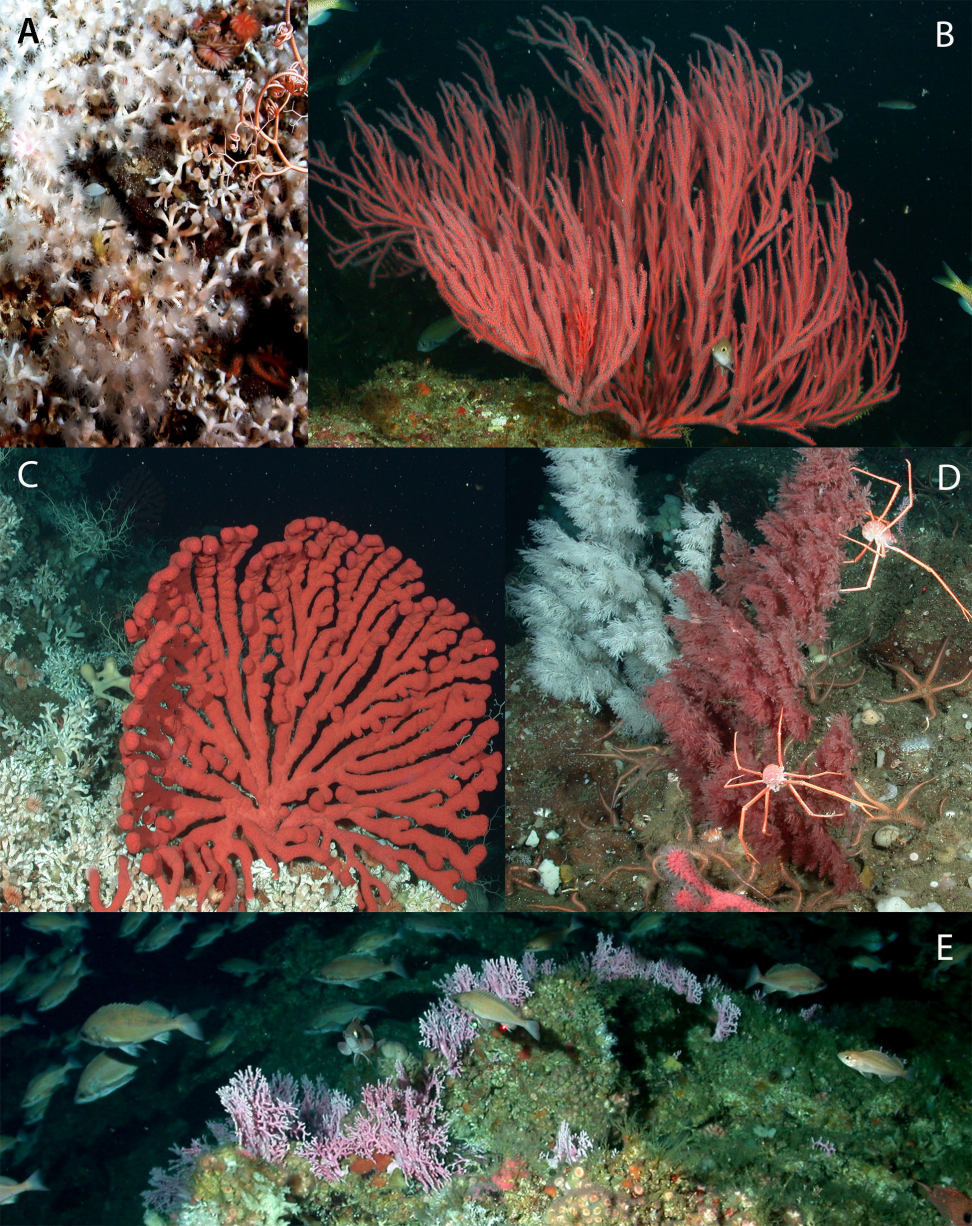
Images depicting structure-forming invertebrates reported. Representative images depicting common structure-forming corals in Southern California. (A) *Lophelia pertusa*, (B) *Leptogorgia chilensis*, (C) *Paragorgia arborea*, and *Lophelia pertusa*, (D) Two color variants of *Antipathes* sp. (E) *Stylaster californicus.* Image source: NOAA.

**Figure 4:**
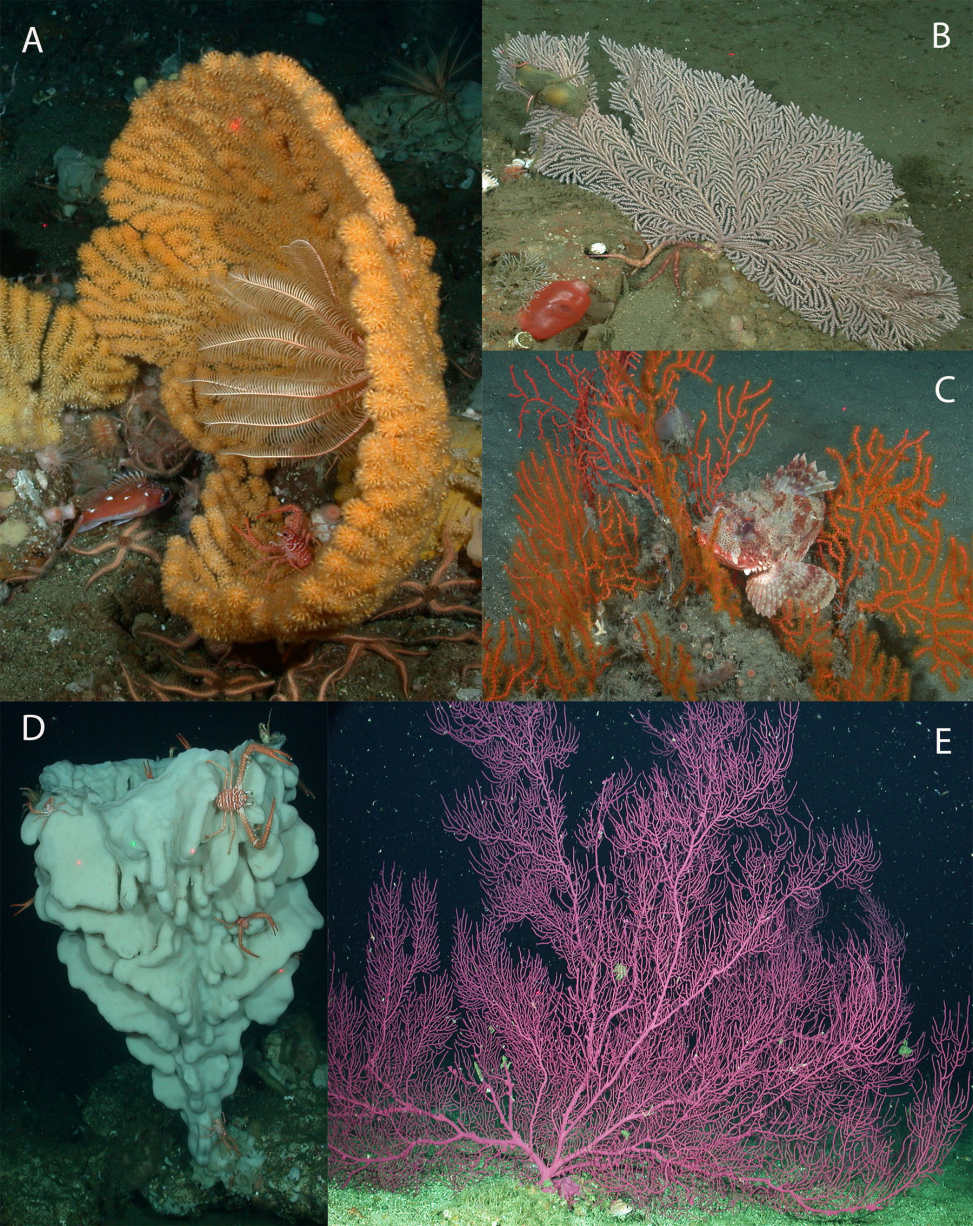
Images depicting structure-forming benthic invertebrates reported. Representative images of common deep-water octocorals in Southern California. (A) *Acanthogorgia* sp., (B) *Plumarella longispina*, (C) *Adelogorgia phyllosclera*, (D) *Aphrocallistes* sp., (E) *Eugorgia rubens*. Lasers are 20 cm. Image source: NOAA.

**Figure 5:**
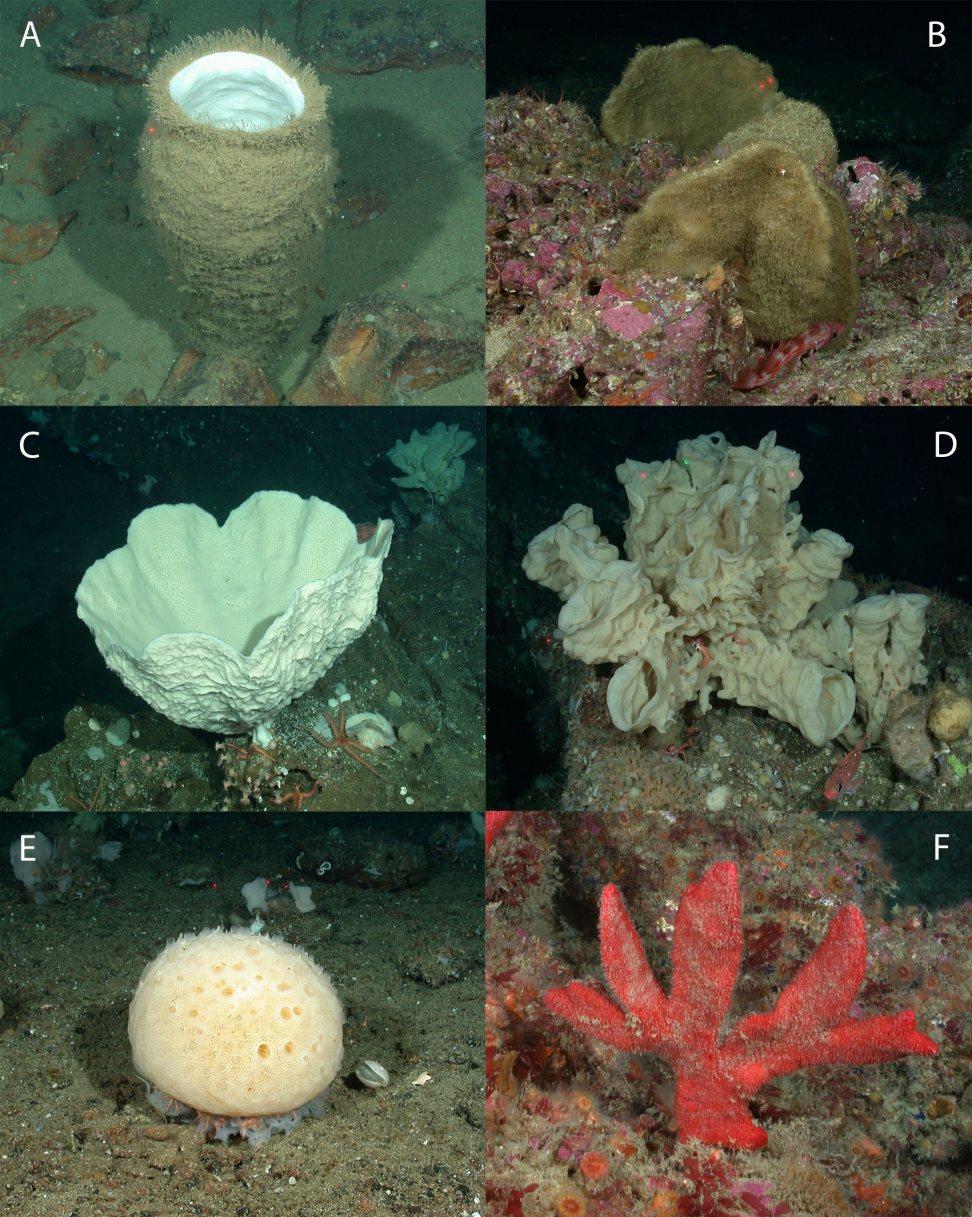
Representative images of the six sponge morphology classifications. Representative images of sponges in Southern California. (A) Barrel (cf. *Acanthascus* sp.), (B) shelf (cf. *Poecillastra* sp.), (C) vase (cf. *Amphilectus* sp.), (D) foliose (cf. *Aphrocallistes* sp.), (E) globular (unidentified genus), and (F) branching (unidentified genus). Image source: NOAA.

Corals were identified from photographs (see Figs. 3 and 4) to the lowest possible taxonomic level using gross morphology and photo reference collections from previously documented specimens from the region. Sponges were classified into seven morphological categories (1–4 adopted from Tissot et al. (2006)): (1) barrel, (2) shelf, (3) vase, (4) foliose, (5) globular, (6) branching, and (7) other (see Figs. 4 and 5). Anthropogenic debris was categorized as: nets, traps, lines and rope, boat gear, and “other refuse,” such as cans, bottles, shoes, dishes.

The size of corals and sponges were recorded as the maximum dimension, height or width, using a laser scale for reference. The corals and sponges were classified into three size categories: 10–20, 21–50, >50 cm. Corals and sponges in each image were classified as “structure-forming” if their height was greater than 10 cm and they exhibited branched morphology; or non-structure-forming (i.e., sea pens, cup corals). The size threshold limited the study to taxa which were large enough to be identified in photos, counted reliably, and potentially subject to impact by bottom fishing gear.

### Coral and sponge community analysis

Richness, evenness, and diversity (Simpson’s index, 1/D) were calculated for each site. Raw abundance (counts) was also documented. The number of transects conducted at the 32 sites varied from one to 40, with a mean of 7.72. The Chao 1 estimator was calculated for each site with seven or more dives using rarefaction analysis in *Estimate S* (Colwell, 2009). A minimum of 10 dives was required to reach asymptote for most sites. Only dives with >30 unique, on-bottom images deeper than 45 m were used. A total of 247 of the 352 transects met these sample size and depth requirements for the diversity analysis.

### Analysis of fishery landings

Landings receipts for commercial fisheries are collected in fishing ports along the coast of California by CDFW. Weight, species, and gear type have been reported and recorded by catch block since 1969 (Fig. S1) and these data have precedent in scientific literature (Miller et al., 2014). Other types of fishing effort data exist from CDFW, such as logbook data, which show start and end points of fishing activity. The data have more precise positioning, but they are confidential. Logbook positions must be averaged over a number of vessels to be shared, compromising the accuracy and precision. Hence, catch blocks for Southern California were used for this analysis, as shown in Fig. S1 (California Department of Fish and Wildlife, 2016).

Commercial landings (pounds) from 2007 to 2011 were categorized by species, year, gear type, and catch block (10 min by 10 min catch blocks, approximately 10.5 × 10.5 miles (17 × 17 km) in dimension, or approximately 280 km^2^ in area) by CDFW’s Marine Fisheries Statistical Unit (Perry et al., 2010; Miller et al., 2014). For this analysis, commercial landings from 209 catch blocks in the SCB (Fig. S1) from 2007 to 2009 were extracted from Perry et al. (2010); commercial landings for 2010–2011 were obtained directly from the California Department of Fish and Wildlife (2011). Landings were filtered to include only data from gear types that targeted deep (>50 m) demersal species, and therefore had the potential to interact with coral and sponge communities. These bottom-contact gear types were classified into four major categories (Table 1): trawls, nets (e.g., gillnets), lines (e.g., bottom longlines), and traps (National Marine Fisheries Service, 2011).

**Table 1:** Bottom-contact fishing gear categories.

Trawls	Nets	Lines	Traps
Bottom trawl	Set gillnet	Set longline	Crab or lobster trap
Single-rigged trawl	Drift gillnet	Monofilament	Fish trap
Trawl net	Tangle net		Prawn trap

**Note:**

Gear categories used in California's deep demersal fisheries.

A total of 70 species codes of the 356 tracked by CDFW represent deep-water demersal species fished by bottom-contact gear (Table 2, see Table S1 for scientific names). Landings of the 70 deep-water demersal fisheries included in this analysis were summed over years and joined with catch block polygons using GIS (ArcGIS 10.3; ESRI, Inc., Redlands, CA, USA). Since not all catch blocks were of equal area, landing values were normalized by dividing total landings per block by catch block area (lbs./km^2^). Landings by catch block and gear type were then visualized using choropleth maps, symbolized by six landings classes defined using Jenks natural breaks (Jenks & Caspall, 1971) for each gear type, for all fixed gear, and for all mobile gear. Commercial landings by catch block were used as an estimator for the distribution and minimum fishing effort throughout the SCB (Miller et al., 2014). Landing data reflect only successful fishing effort, and not true fishing effort.

**Table 2:** List of landings categories.

Landing categories			
Hagfishes	Rockfish, darkblotched	Rockfish, bolina	Shark, spiny dogfish
Lingcod	Rockfish, flag	Rockfish, deep-water red	Shrimp, ocean (pink)
Prawn, ridgeback	Rockfish, greenblotched	Rockfish, red	Sole, butter
Prawn, spot	Rockfish, greenspotted	Rockfish, rosefish	Sole, Dover
Rockfish, aurora	Rockfish, greenstriped	Rockfish, shelf	Sole, English
Rockfish, bank	Rockfish, pinkrose	Rockfish, slope	Sole, fantail
Rockfish, black	Rockfish, redbanded	Rockfish, small	Sole, petrale
Rockfish, blackgill	Rockfish, rosethorn	Rockfish, Mexican	Sole, rex
Rockfish, bocaccio	Rockfish, rosy	Rockfish, olive	Sole, rock
Rockfish, bronzespotted	Rockfish, speckled	Rockfish, Pacific ocean perch	Sole, sand
Rockfish, brown	Rockfish, splitnose	Rockfish, pink	Sole, tongue
Rockfish, canary	Rockfish, squarespot	Rockfish, widow	Sole, unspecified
Rockfish, chameleon	Rockfish, starry	Rockfish, yelloweye	Thornyhead, longspine
Rockfish, chilipepper	Rockfish, stripetail	Rockfish, yellowtail	Thornyhead, shortspine
Rockfish, China	Rockfish, swordspine	Sablefish	Thornyheads
Rockfish, copper	Rockfish, unspecified	Sea cucumber, giant red	Trawled fish for animal food
Rockfish, copper (whitebelly)	Rockfish, vermilion	Sea cucumber, unspecified	
Rockfish, cowcod	Rockfish, bocaccio/chili	Shark, soupfin	

**Note:**

List of landings categories from deep-water (>50 m) demersal fisheries used to calculate demersal landings in the Southern California Bight.

### Spatial overlap between commercial fisheries and deep-water corals and sponges

Spatial overlap between deep-water demersal fisheries and deep-water corals is considered here in three forms: co-occurrence in images of the seafloor; depth range overlap (coincident depth range); and geographic overlap, or coincident latitudinal and longitudinal range. We used ROV images to quantify the number of co-occurrences between fishing gear and coral and sponge fauna. For the purposes of this study, a co-occurrence was defined as the presence of fishing gear debris in the same image as structure-forming corals or sponges. Anthropogenic debris was identified as one of five types: fishing nets, fishing traps, fishing lines, other discarded boating-related gear, or miscellaneous trash. Only the first three debris types were associated with fishing activity.

Occurrences were used to examine the depth distributions of predominant corals and sponges in relation to the depth distribution of fishing debris, to examine potential interactions. Since the depths of observations were not normally distributed, a Kruskal–Wallis one-way analysis of variance was used to determine whether the distributions of corals and fishing debris was statistically different. Post hoc, pairwise analysis using Mann–Whitney tests was performed on statistically different depth ranges. All statistical analyses were conducted using R software (R Core Team, 2016).

Visualization of depth distribution was performed using smoothed density curves generated by an algorithm in R software (R Core Team, 2016). The algorithm seeks to estimate an optimal model according to Bayesian information criterion for expectation maximization initialized by hierarchical clustering for parameterized Gaussian mixture models. The approach uses hierarchical clustering to estimate the optimal number of clusters based on the different distributions that make up the overall density structure (Fraley, Raftery & Scrucca, 2012). The estimation assumes that sampling effort is adequate to reflect the true distribution(s).

The potential geographic overlap of fisheries and invertebrates was determined by overlaying coordinates of corals, sponges, and fishing debris onto maps of CDFW landings data using GIS. Depth, latitude, and longitude of ROV observations were used to plot the geographic and depth distribution of structure-forming invertebrate taxa and fishing debris. ROV observations were plotted over CDFW fishing blocks in GIS to identify the depths and regions (i.e., catch blocks) where interactions between corals, sponges, and bottom-contact fishing gear are likely to occur.

### RAFi index

We used findings from our analyses of coral and sponge communities, fisheries landings, and gear observations to create an index of co-occurrence between deep-water benthic communities and fisheries within the SCB. The index included parameters for richness, abundance, frequency, and fishing intensity. Summary scores for the index, which we called RAFi, were calculated by assigning normalized values (0.00–1.00) for each parameter, and summing the four parameters of richness, abundance, frequency, and fishing intensity equally to yield a maximum possible score (4.00) for each site as follows. The richness “R” parameter used Simpson’s reciprocal mean (1/D). The abundance parameter “A” had two types of values: counts of corals and sponges (raw abundance); and frequency of coral and sponges (normalized abundance). In this way, a cumulative value was captured for the total survey extent, as well as a relative value that incorporates differing degrees of search effort. The fishing intensity “Fi” parameter was calculated as the sum of the landings rank (0–5) determined by Jenks natural breaks, plus the normalized frequency of fishing gear observations per ROV image, to yield a maximum score of 1.00.

As an example, consider a study site with the highest diversity of a Simpson’s reciprocal mean value of 9.2 (Piggy Bank). Dividing by this maximum value across all sites generates a maximum Richness (R) score of 1.00, and a minimum score of 0.22 (Mission Beach Reef). This process, where *score = value × 1/maximum value*, was repeated for the other parameters. Abundance, frequency, and fishing intensity scores had two equally weighted components with maximum subscores of 1.0, where *score* = [(*subscore A + subscore B*)/2]. Subscore values for corals and sponges ranged from 0 to 1.0, respectively. These added to a maximum score of 2.0, subsequently divided by 2, to yield a maximum of 1.0. Fishing intensity consisted of a “gear score” and a “landings score.” These were also equally-weighted with maximum values of 1.0. The gear score for the study site was based upon the percentage of images that contained fishing gear. The landings score was based on the rank (0–5) of the landings category, divided by 5, to yield values on a scale of 0.0–1.0. Gear scores and landings scores were added together to generate a Fishing intensity (Fi) score ranging from 0.04 (corresponding to a low percentage of images with gear during ROV dives at that site and low reported landings for the block encompassing that site) to 0.97 (corresponding to a high frequency of gear in ROV images at the site and relatively high degree of reported landings). Finally, summary values for the RAFi index were calculated as follows:

$${\rm{RAFi}}\left( {{\rm{0-4}}} \right)= {\rm{ R \, score }}\left( {{\rm{0-1}}} \right)+ {\rm{ A \, score }}\left( {{\rm{0-1}}} \right)+ {\rm{ F \, score }}\left( {{\rm{0-1}}} \right)+ {\rm{ Fi \, score }}\left( {{\rm{0-1}}} \right)$$

The final RAFi scores were then ranked to yield the level of priority. A number of iterations took place. Two sets of RAFi tables were calculated, one for all structure-forming taxa, and another for “vulnerable” taxa only, as defined by those taxa in which more than 5% of observations are in the maximum size category, >50 cm. The index was also calculated without fishing intensity values, to determine which sites hosted high diversity and abundance of corals and sponges, regardless of fishing. A map was created once RAFi ranks were determined for sites, and catch blocks were assigned a priority level from high to low (Fig. S1). The levels assigned were: high priority (high landings and many corals or sponges); medium priority (moderate landings and some corals or sponges, or moderate landings with no ROV survey); or low priority (low landings, no evidence of corals or sponges in ROV surveys, or both).

## Results

### Abundance and diversity of coral and sponge observations

Of the 352 dives analyzed, 331 contained benthic images deeper than 45 m. A total of 26,759 unique photos were analyzed, with 23% containing corals or sponges (6,051). These photos contained 9,610 occurrences (incidences) of corals and/or sponges (Table 3). Scleractinian corals occurred in 1,166 unique images, or 4.4% for a total of 2,060 colonies. *Desmophyllum* and *Coenocyathus* were the most commonly observed. The branching scleractinian *Lophelia pertusa* occurred in 230 images (1%) with a total of 568 colonies counted. The branching hydrocoral *Stylaster californicus* was observed in 172 images, and 693 colonies were counted. Gorgonian octocorals were the most common corals, they occurred in 2,301 unique images, or 8.6% with a total of 5,136 observed colonies. Sponges were more common than corals. Sponges were observed in 5,736 images (21%), and 12,037 sponges were counted (Table 3).

**Table 3:** Summary of photo observations.

Images reviewed	34,792
Duplicate or non-benthic images	8,033
Unique benthic images	26,759
Photos with corals or sponges	6,051

Occurrences	Photos	Frequency	Colonies	Composition (%)
**3D sponges**	**5,736**	**0.21**	**12,037**	**59.56**
Foliose	1,439	0.05	4,015	19.87
Shelf	1,328	0.05	2,505	12.40
Barrel	1,118	0.04	2,089	10.34
Vase	1,000	0.04	2,011	9.95
Globular	586	0.02	958	4.74
Branching	169	0.01	345	1.71
Other sponges	96	0.00	114	0.56
**Scleractinia**	**1,166**	**0.04**	**2,060**	**10.19**
*Desmophyllum*	576	0.02	823	4.07
*Lophelia*	230	0.01	568	2.81
*Coenocyathus*	290	0.01	550	2.72
*Dendrophyllia*	70	0.00	119	0.59
**Stylasteridae**	**172**	**0.01**	**693**	**3.43**
*Stylaster*	172	0.01	693	3.43
**Gorgonacea**	**2,301**	**0.09**	**5,136**	**25.41**
*Adelogorgia*	535	0.02	1,882	9.31
*Eugorgia*	436	0.02	792	3.92
*Leptogorgia*	389	0.01	615	3.04
*Acanthogorgia*	150	0.01	409	2.02
*Plumarella*	235	0.01	402	1.99
*Swiftia*	175	0.01	225	1.11
*Paragorgia*	137	0.01	149	0.74
*Muricea*	37	0.00	64	0.32
*Parastenella*	7	0.00	7	0.03
Other Gorgonacea	200	0.01	591	2.92
**Antipatharia**	**235**	**0.01**	**283**	**1.40**
*Antipathes*	235	0.01	283	1.40
**Total Occurrences**	**9,610**	**0.23**	**20,209**	**100.00**

**Note:**

Summary of all photo observations of deep-water corals and sponges in the Southern California Bight.

Bold text indicates category totals.

**Figure 6:**
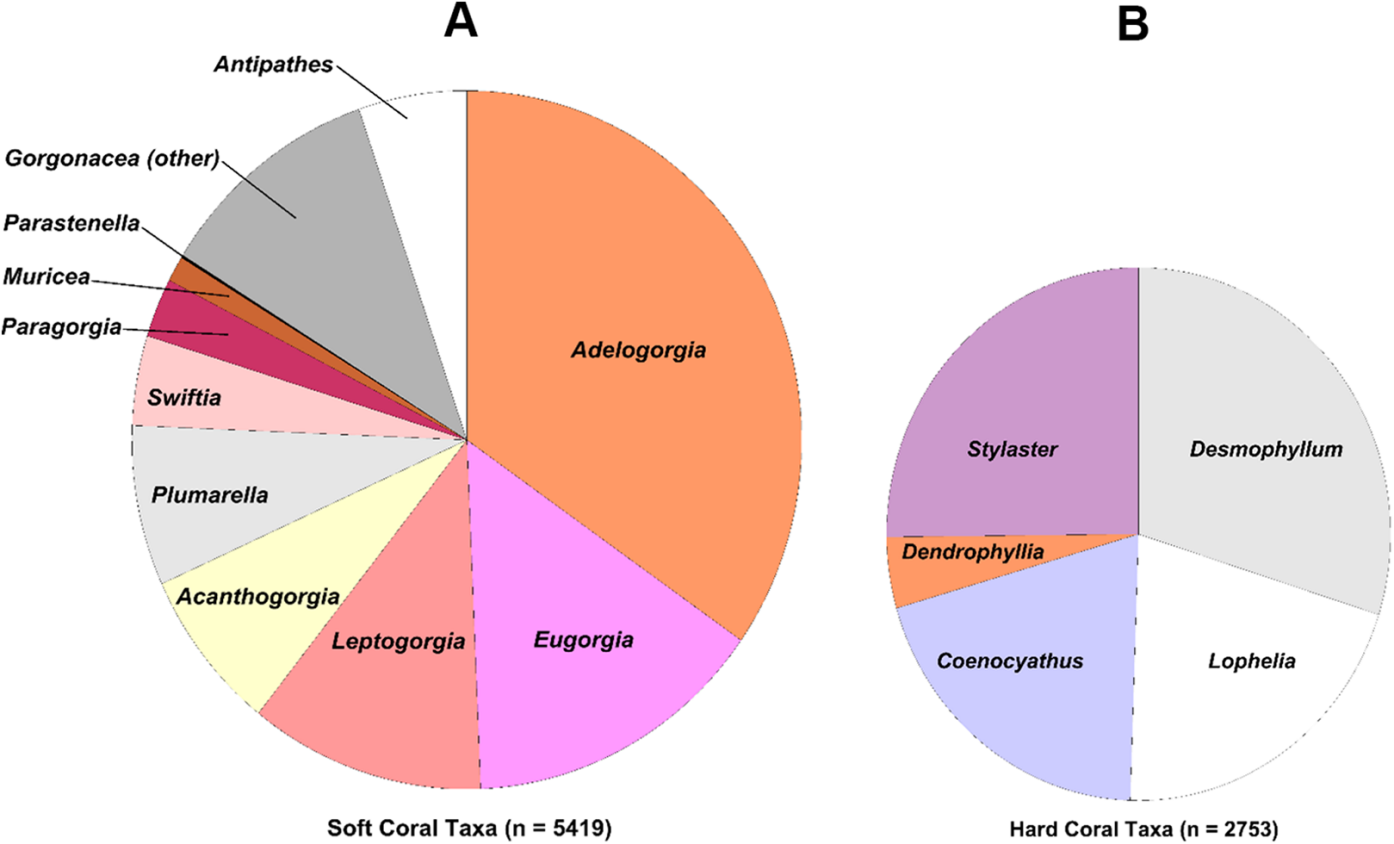
Pie charts showing percent observation counts. Pie charts showing percent observation counts of soft coral genera ((A), *n* = 5,419) and hard coral genera ((B), *n* = 2,753).

**Figure 7:**
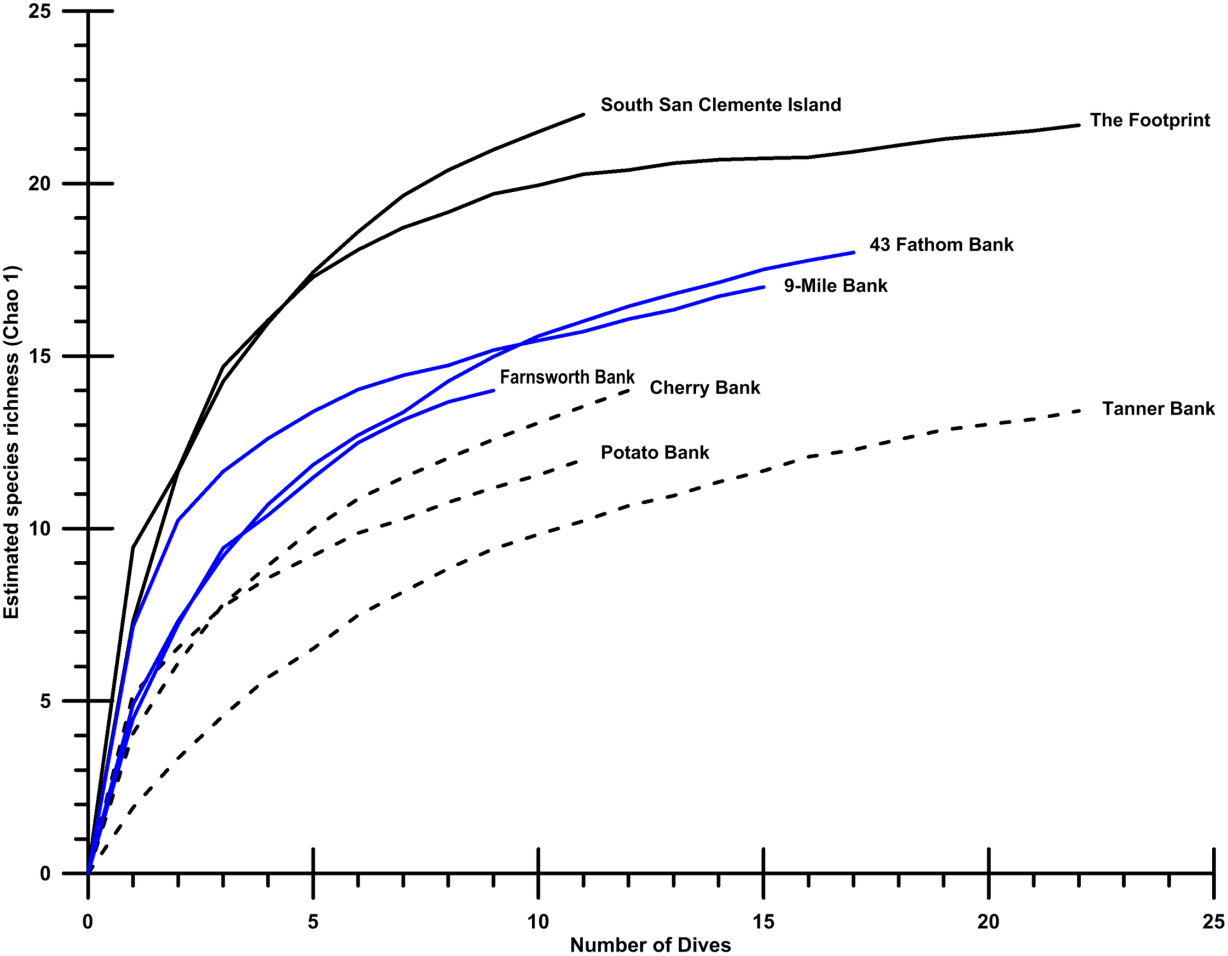
Species accumulation plots. Species accumulation plots showing Chao 1 richness for eight localities surveyed with at least 10 dives containing 30 observations, or more. Of the study sites shown, The Footprint, Cherry Bank, Potato Bank, and 43 Fathom are essential fish habitat (EFH) areas. Southern San Clemente Island, 9-Mile Bank, Tanner Bank, and Farnsworth Bank are outside current EFH.

The coral and sponge assemblages had high richness and evenness (Pielou’s J’ range of 0.5–1.0). Figure 6 shows the relative proportions of observation for each genus of soft and hard. The number of coral genera and sponge morphotypes found in each study site varied from a minimum of six taxa, in East Catalina Island, to a maximum of 22 taxa, in The Footprint. Since the number of dives in each site varied as well, we used Chao 1 species richness to illustrate diversity at sites where seven or more dives were conducted (Fig. 7). The ROV survey data shows some sites reach 7–10 genera in seven dives, while others rise to 18–20 different genera in seven dives. South San Clemente Island and The Footprint show the greatest rates of accumulation for coral and sponge taxa (>20); 43 Fathom, 9-Mile, and Farnsworth Bank had moderately high richness (15–19); and Potato, Cherry, and Tanner Banks exhibited the lowest number of taxa (10–14).

**Figure 8:**
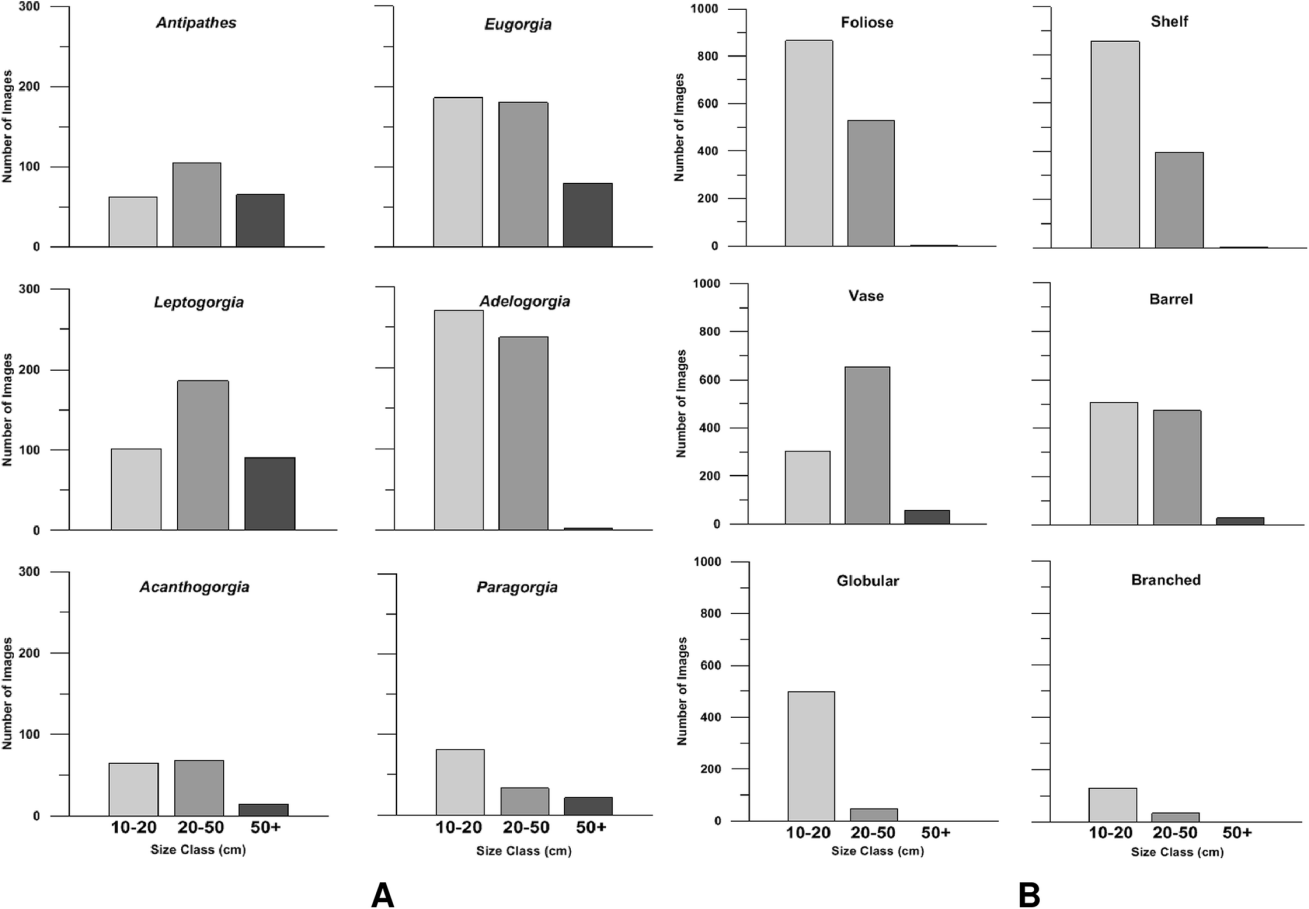
Size class distributions. Bar charts of the size class distribution for predominant deep-sea corals (A) and sponge morphotypes (B) in Southern California.

In general, soft corals comprised the largest size class (>50 cm, Fig. 8A). Gorgonian octocorals *Leptogorgia chilensis* and *Eugorgia rubens*, and the black coral *Antipathes dendrochristos* commonly exceeded 50 cm in size. *Acanthogorgia* and *Paragorgia* also exceeded this threshold. All the gorgonian octocorals had a large proportion of colonies in the 21–50 cm size class as well. Among the sponges, the vase-shaped and barrel-shaped morphotypes (e.g., *Aphrocallistes* and *Acanthascus*) occasionally grew larger than 50 cm size. The most common morphotypes, foliose and shelf sponges, were generally in the size classes 10–20 and 21–50 cm (Fig. 8B).

### Number and types of fishing debris observations

Fishing debris was relatively rare compared to corals and sponges. A total of 914 occurrences of anthropogenic debris (3.4% of photos) were identified, including: 364 images of lines or rope, 292 images of other refuse, 183 pieces of boat gear (all types), 52 images of nets, and 23 images of traps (Table 4). Lines and rope were the most commonly observed anthropogenic debris. “Lines” includes monofilament lines and “rope” includes polypropylene ropes and other types.

**Table 4:** Anthropogenic debris types.

Debris type	Observations	Percent of images (%)	Percent of total debris (%)
Lines/rope	364	1.36	39.82
Other refuse	292	1.09	31.95
Boat gear	183	0.68	20.02
Nets	52	0.19	5.69
Traps	23	0.09	2.52
Total debris	914	3.41	100.00

**Note:**

Anthropogenic debris in the Southern California Bight survey area by debris type.

### Co-occurrences between bottom-contact fishing gear and corals or sponges

**Figure 9:**
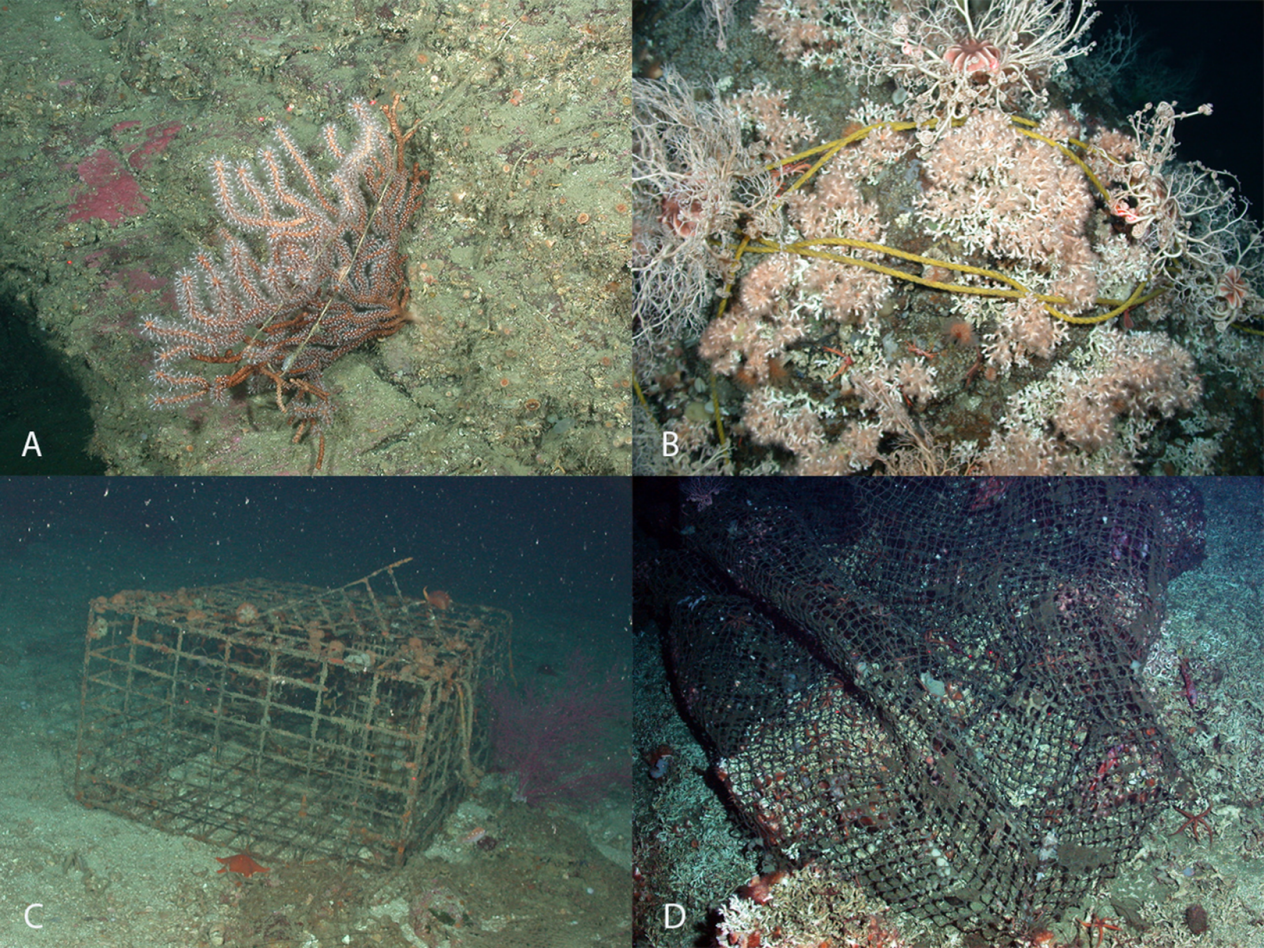
Interactions between fishing debris and deep-water coral in Southern California Bight. (A) Bottom set line with *Muricea* sea fan, (B) a polypropylene line around *Lophelia pertusa*, (C) a trap and net adjacent to *Eugorgia* sea fan, and (D) trawl netting around *Lophelia pertusa.* Image source: NOAA.

A total of 294 co-occurrences of fishing gear and corals or sponges were observed in 914 photos (Table 5). Occurrences with lines/rope were most prevalent (*n* = 208). Figure 9 shows example images depicting co-occurrences, where fishing gear and benthic fauna were observed. Most co-occurrences involved corals (*n* = 127), but many 3D sponges (*n* = 116) also co-occurred with fishing gear. Boat gear were the second most prevalent debris type (*n* = 54), followed by nets (*n* = 24) and traps (*n* = 8). Although sponge observations were more frequent than coral observations, co-occurrences with fishing gear were more common for corals (Table 5).

**Table 5:** Co-occurrence of biota and fishing gear.

	Traps	Nets	Lines/rope	Boat gear	Total
Corals	6	14	127	31	178
Sponges	2	10	81	23	116
Total	8	24	208	54	294

**Note:**

Summary of anthropogenic debris types observed as co-occurring with corals and/or sponges.

### Vertical distribution of corals and sponges

**Figure 10:**
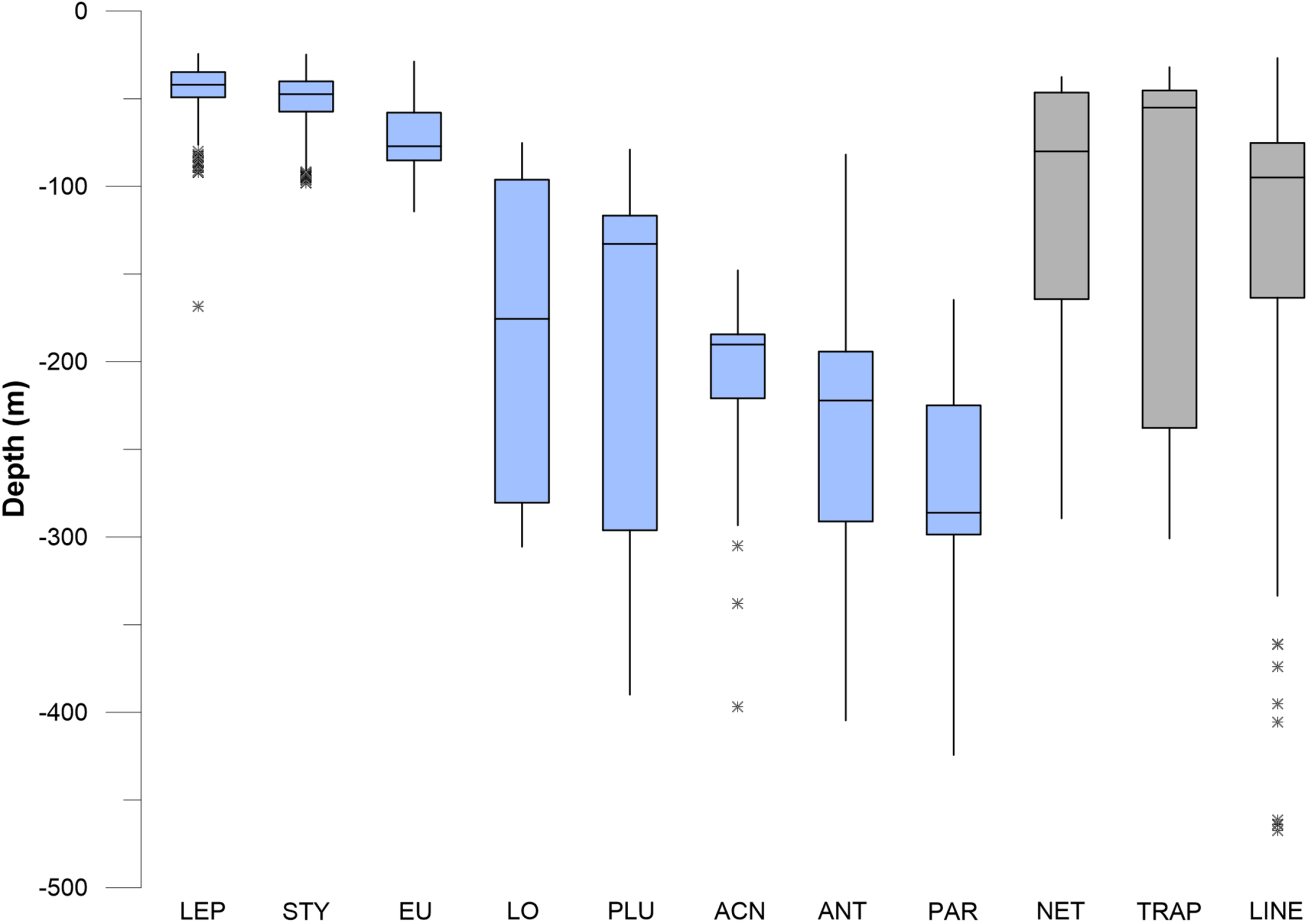
Box and whisker plots of depth values for fishing debris and corals. The plots show minimum and maximum values as vertical lines, first and third quartiles as a box, and median values as a horizontal line. Asterisks (*) are outliers (>1.5 interquartile range). Abbreviations are: *Leptogorgia chilensis* (LEP, *n* = 389), *Stylaster californicus* (STY, *n* = 172), *Eugorgia rubens* (EU, *n* = 436), *Lophelia pertusa* (LO, *n* = 230), *Plumarella* sp. (PLU, *n* = 235), *Acanthogorgia* sp. (ACN, *n* = 150), *Antipathes* sp. (ANT, *n* = 235), and *Paragorgia* sp. (PAR, *n* = 137). Sample sizes for debris are: traps (*n* = 23), nets (*n* = 52), lines (*n* = 364).

**Figure 11:**
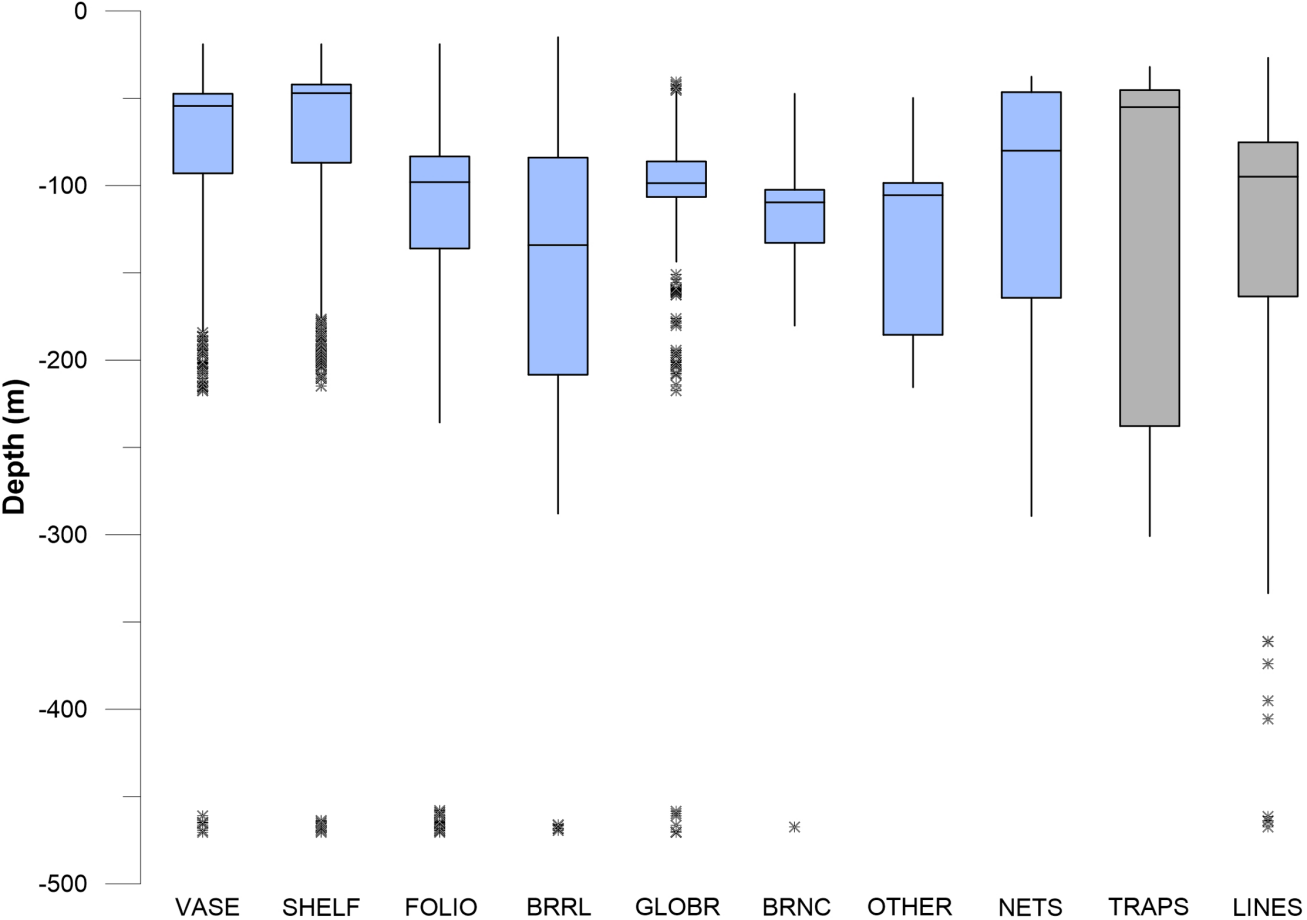
Box and whisker plots of depth values for fishing debris and sponges. Depth values for seven sponge morphotypes are shown with three different gear types. Asterisk * indicates outlier.

Corals and sponges occurred throughout the vertical (depth related) range of 45–475 m depth, but there were significant differences in the depth ranges of individual taxa (Kruskal–Wallis, *p* < 0.05), as shown in the box plots in Figs. 10 and 11. Corals were stratified into two general groups: a “shallow” (50–100 m) group and a “deep” (100–300 m) group (Fig. 10). *Stylaster, Leptogorgia*, and *Eugorgia* were commonly observed in the 50–100 m depth range. Deep gorgonian genera (>100 m) were most frequently represented by the genera *Plumarella*, *Acanthogorgia*, *Antipathes*, and *Paragorgia*. The “shallow” group also included *Adelogorgia phyllosclera*, with a depth range of 30–128 m and a median value of 83 m.

In pairwise comparisons between shallow taxa, no statistical difference was found between depth ranges of *Stylaster*, *Eugorgia*, and *Leptogorgia* (Kruskal–Wallis, *p* > 0.05). The depth ranges of *Stylaster*, *Eugorgia*, and *Leptogorgia* were significantly different than those of the “deep” group; consisting of the scleractinian *Lophelia pertusa*; the black coral *Antipathes* and the gorgonians *Plumarella*, *Acanthogorgia*, and *Paragorgia* (Kruskal–Wallis, *p* > 0.05 and Mann–Whitney *Z* > 1.96). The overall depth ranges for the “deep” group were from 70 to 400 m, with median values from 125 to 275 m and third quartiles reaching to 300 m (Fig. 10). “Bubblegum” octocorals in the genus *Paragorgia* had the deepest median range and had a statistically different (Mann–Whitney *Z* > 1.96) depth range than *Lophelia* and *Plumarella*, which extend into depths as shallow as 100 m. Sponges occupied a broad depth range, with a minimum depth of 30 m, a maximum depth of 470 m, and a median value near 150 m (Fig. 11).

### Vertical distribution of fishing debris

Debris was identified in still images throughout the depth range of photos, from 27 to 470 m. Median values for traps, nets, and lines/rope were 50–100 m, with third quartiles of traps reaching to 230 m and third quartiles for nets and lines/rope reaching to 150 m (Figs. 10 and 11). Lines and rope had the deepest occurrences among gear types. There was no significant difference in depth range among fishing gear debris types (Kruskal–Wallis, *p* > 0.05). The highest concentration of debris was generally from 50 to 250 m depth. Survey effort by the ROV was also highest in this range. Outliers suggest that lines and ropes are likely to occur in deeper waters. Outliers were defined as >1.5 times the interquartile range. These outliers fell between 350 and 500 m depth (Figs. 10 and 11). There was one outlier at 568 m, a single observation of monofilament line.

### Depth range overlap between benthic communities and fishing debris

**Figure 12:**
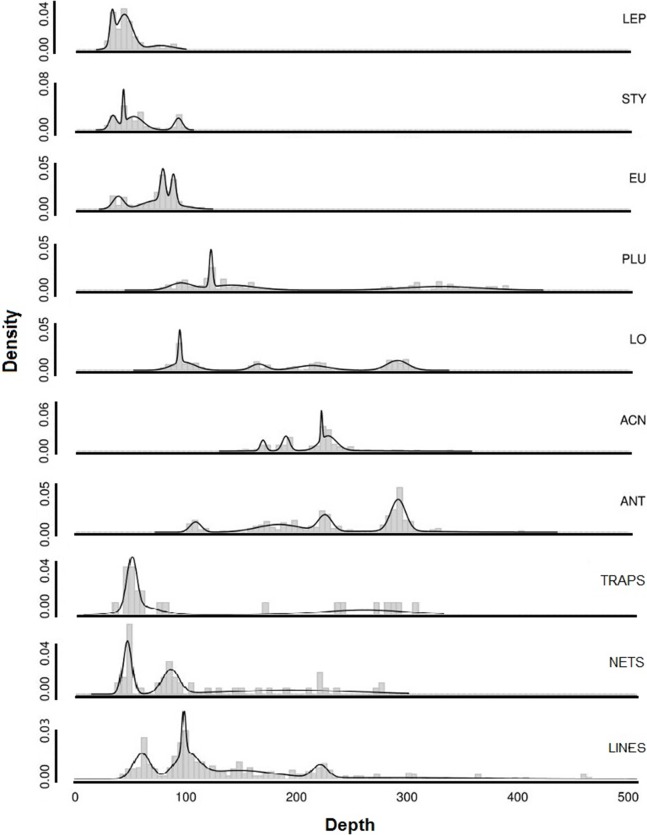
Smoothed density distributions curves for most common genera of deep-sea corals in Southern California. Abbreviations are: *Leptogorgia chilensis* (LEP, *n* = 389), *Stylaster californicus* (STY, *n* = 172), *Eugorgia rubens* (EU, *n* = 436), *Lophelia pertusa* (LO, *n* = 230), *Plumarella* sp. (PLU, *n* = 235), *Acanthogorgia* sp. (ACN, *n* = 150), *Antipathes* sp. (ANT, *n* = 235), and *Paragorgia* sp. (PAR, *n* = 137). Sample sizes for debris are: traps (*n* = 23), nets (*n* = 52), lines (*n* = 364).

The depth ranges of predominant shallow genera (*Leptogorgia*, *Eugorgia*, and *Stylaster*) were statistically different from the depth ranges of predominant deep genera (*Lophelia*, *Acanthogorgia*, *Plumarella*, and *Antipathes*) in pairwise Kruskal–Wallis comparisons (*p* < 0.05). No significant difference was found between depth ranges of fishing gear debris type observations (Kruskal–Wallis, *p* > 0.05). The depth ranges were statistically similar (*p* > 0.05) between three predominant coral taxa (*Stylaster*, *Leptogorgia*, and *Eugorgia*) and observations of fishing debris. Corals with deeper ranges did not have statistically equivalent depth ranges with fishing gear debris, although co-occurrences were observed as deep as 500 m. Smoothed density distribution visualizations of predominant taxa and fishing gear are shown on Fig. 12. The histograms for fishing gear show peaks in the same depth range as coral genera *Leptogorgia*, *Stylaster*, *Eugorgia*, and *Lophelia*.

### Geographic distribution of corals and sponges in the SCB

**Figure 13:**
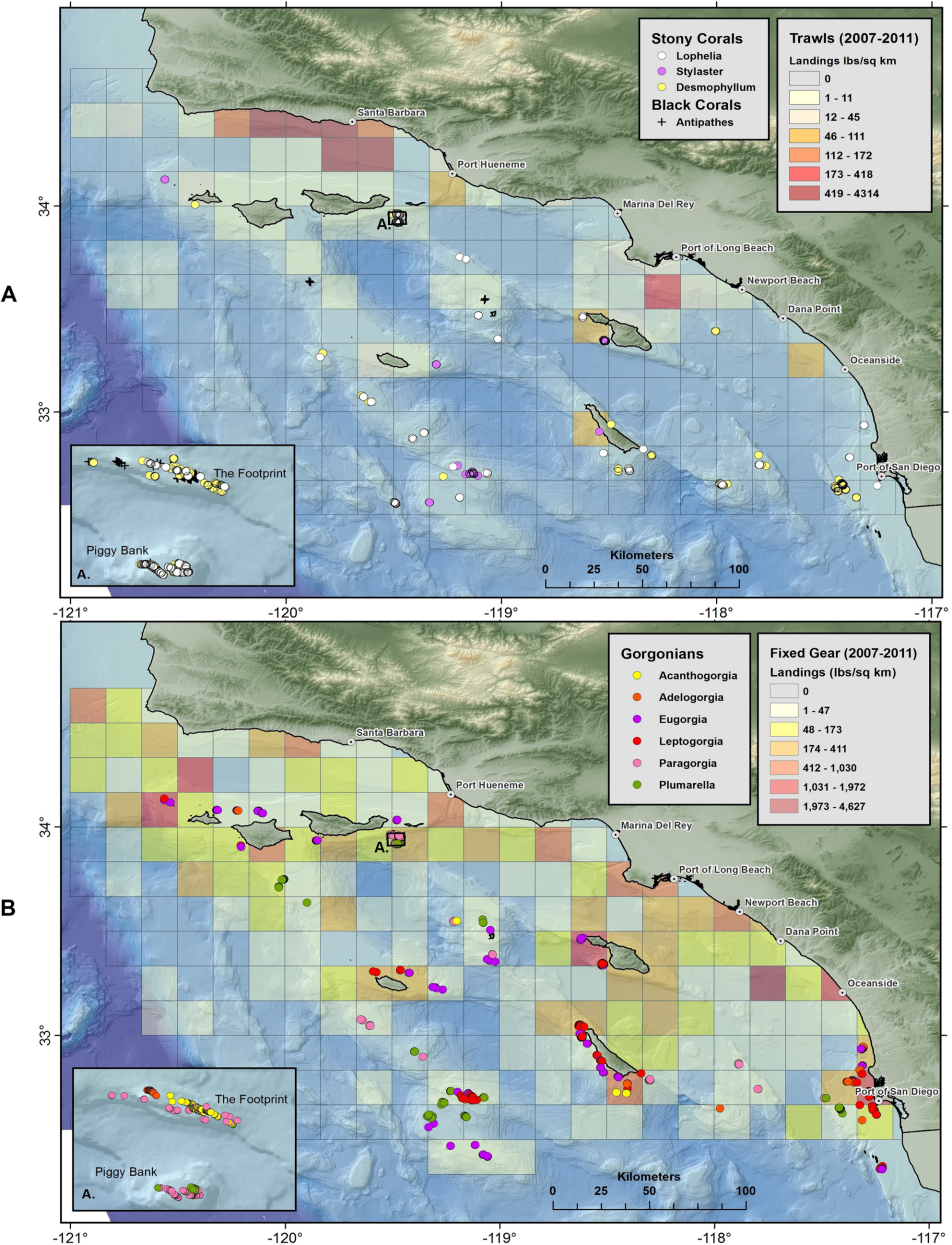
Maps showing geographic distribution of commercial demersal fisheries landings in the Southern California Bight. (A) Mobile bottom-contact fishing gear (trawls), with Scleractinia and Stylaster; and (B) Fixed bottom-contact fishing gear (lines, nets, and traps) with gorgonians (Alcyonacea). Reprinted from Rooper et al. (2017).

Maps of stony corals show that scleractinian and lace corals are broadly distributed on offshore banks and mounds in Southern California (Fig. 13A). Gorgonians (Fig. 13B) and sponges (Figs. S2 and S3) are also widespread throughout the 50–500 m depth range. Aggregations of corals occur in high abundance in several areas, namely Piggy Bank, The Footprint, San Clemente Island, Santa Catalina Island, Cortes Bank, and Tanner Bank, among others.

### Fisheries using bottom-contact gear in the SCB and geographic distribution of landings

A table of landings by gear type was compiled to identify the “top 10” deep-water demersal fisheries by weight, landed in the SCB for the time periods of 1972–2011 and 2007–2011 (Perry et al., 2010; California Department of Fish and Wildlife, 2011; Table 6). Of the 70 species codes identified as deep-water demersal species, six (sablefish, shortspine thornyhead, ridgeback prawn, hagfish, spot prawn, sea cucumber) accounted for 93% of landings since 2007 (Table 6). Demersal gear accounts for 69–100% of the total reported landings of the top 10 species during this period (Table 6, column 5). Bocaccio, blackgill rockfish, and red rockfish are also taken by gear which typically targets pelagic species.

**Table 6:** Top 10 Landings.

2007–2011	Rank	Total landings (lbs.)	Demersal gear (%)	Total by weight (%)	Set lines (%)	Set nets (%)	Traps (%)	Trawls (%)
Sablefish	1	1,974,141	96	20	93	0	7	0
Thornyhead, shortspine	2	1,718,206	99	18	100	0	0	0
Prawn, ridgeback	3	1,699,840	99	18	0	0	0	100
Hagfishes	4	1,390,398	100	14	0	0	100	0
Prawn, spot	5	1,149,301	100	12	0	0	99	1
Sea cucumber, giant red	6	1,065,838	94	11	0	0	0	100
Thornyhead, longspine	7	186,266	97	2	100	0	0	0
Sole, unspecified	8	139,084	97	1	1	1	0	98
Rockfish, blackgill	9	118,797	69	1	95	1	4	0
Thornyheads	10	44,187	99	0	100	0	0	0
Total		9,486,058		98				

1972–2011	Rank	Total landings (lbs.)	Demersal gear (%)	Total by weight (%)	Set lines (%)	Set nets (%)	Traps (%)	Trawls (%)
Sablefish	1	20,802,653	95	28	22	0	77	1
Rockfish, unspecified	2	14,096,308	42	19	10	74	1	16
Prawn, ridgeback	3	8,625,517	94	12	0	0	0	100
Prawn, spot	4	4,072,476	89	5	0	0	74	26
Rockfish, bocaccio	5	3,279,164	52	4	6	55	0	38
Sea cucumber, unspecified	6	3,266,302	45	4	0	0	0	100
Thornyhead, shortspine	7	3,141,487	99	4	98	0	1	1
Rockfish, bocaccio/chili	8	2,043,290	67	3	1	99	0	1
Sea cucumber, giant red	9	2,020,026	97	3	0	0	0	100
Rockfish, group red	10	1,767,640	40	2	63	35	0	2
Total		63,114,863		84				

**Note:**

Summary of landings for the “top 10” deep, demersal species in the Southern California Bight by gear type.

Approximately 9.5 million pounds of seafood were removed from the deep seafloor in the SCB between 2007 and 2011, according to this analysis. Sablefish (*Anoplopoma fimbria*) and shortspine thornyhead (*Sebastolobus alascanus*) had the highest landings (Table 6), primarily from bottom set lines. These two fisheries account for 38% of all demersal landings between 2007 and 2011. Landings of ridgeback prawn (*Sicyonia ingentis*), red sea cucumber (*Parastichopus californicus*), and sole (primarily in the family Soleidae, but with some Pleuronectidae and Paralichthyidae) were from bottom trawls and account for 30% of total landings. Hagfish (*Eptatretus stoutii*), spot prawn (*Pandalus platyceros*), and rockfish (genus Sebastes) were taken by traps and represented about 27% of total landings.

Three spatial patterns are evident in plots of demersal fisheries landings from 2007 to 2011. First, landings by bottom trawl fisheries appear to be concentrated in the Santa Barbara Channel region and near the Port of Long Beach, with a few catch blocks reporting landings from San Nicolas, Santa Catalina, and San Clemente Islands (Fig. 13A). Second, fixed gear fisheries (set lines, traps, set nets) appear to have a much broader geographic range in the SCB compared to mobile gear (Fig. 13B and Figs. S2–S4). Landings for fixed gear fisheries are reported from 147 catch blocks compared to 52 catch blocks for bottom trawl fisheries. Third, the geographic footprint of fixed gear fisheries extends farther offshore than bottom trawl fisheries, especially south and west of the Channel Islands. The mean distance from shore (closest point) for catch blocks with landings larger than 50 lbs./km^2^ was 16.4 km (σ = 9.2) for trawl landings, while the mean distance for fixed gear landings was 41 km (σ = 28.2) from shore.

### RAFi indices

The RAFi indices effectively identified sites with high abundance and diversity for corals and sponges, and some evidence of fishing activity. The top 10 ranked sites were robust when fishing scores were excluded, but changed when fishing was included, to up-rank some sites and down-rank others. Indices were examined using the combinations—richness and abundance (without frequency); richness and frequency (without abundance); richness, abundance, and frequency (without fishing components); and the full complement richness, abundance, fishing intensity.

The RAFi index scores were based primarily on the numbers of corals and sponges. The total counts are listed by taxon and by bank in supplemental material tables (Tables S2 and S3). Piggy Bank and The Footprint had highest total abundance of corals, and Cherry Bank had high abundance of sponges. Summary scores yielded 32 sites (localities) that were ranked (Tables 7 and 8). Result scores from richness, abundance, and frequency values without fishing components are shown in Table 9.

**Table 7:** RAFi All taxa.

All taxa	Observations		Occurrences			Colonies		Richness			Abundance			Frequency			Fishing Intensity					*RAFi*	
Locality	*n*	Transect count	Gear	Coral occurrences	Sponge occurrences	Coral total	Sponge total	Total taxa	Simpson’s 1/D	R score*	Abundance CO	Abundance SP	A score*	Coral ratio	Sponge ratio	F score*	Gear ratio	Gear score	Land	Land score	FI score*	Sum	Rank
Piggy Bank	736	6	9	485	625	978	1,052	17	9.2	**1.00**	0.75	0.27	**0.51**	0.66	0.85	**1.00**	0.01	0.12	3.0	0.60	0.36	**2.87**	**1**
The Footprint	2,170	26	34	651	1,456	1,304	3,906	22	6.21	**0.68**	1.00	1.00	**1.00**	0.30	0.67	**0.64**	0.02	0.15	3.0	0.60	0.38	**2.69**	**2**
W Santa Catalina Island	195	2	19	176	4	371	12	13	4.67	0.51	0.28	0.00	0.14	0.90	0.02	**0.61**	0.10	0.94	5.0	1.00	**0.97**	**2.23**	**3**
Farnsworth Bank	643	9	67	220	17	551	23	14	4.05	0.44	0.42	0.01	**0.21**	0.34	0.03	0.24	0.10	1.00	4.0	0.80	**0.90**	**1.80**	**4**
Del Mar Steeples	202	4	14	184	13	490	26	13	2.22	0.24	0.38	0.01	**0.19**	0.91	0.06	**0.65**	0.07	0.67	3.0	0.60	**0.64**	**1.71**	**5**
S San Clemente Island	932	11	13	97	168	130	272	22	8.73	**0.95**	0.10	0.07	0.08	0.10	0.18	0.19	0.01	0.13	4.0	0.80	**0.47**	**1.69**	**6**
109 Seamount	268	1	19	69	167	181	313	14	5.64	0.61	0.14	0.08	0.11	0.26	0.62	0.58	0.07	0.68	0.0	0.00	0.34	**1.65**	**7**
9-Mile Bank	1,569	15	30	200	278	258	351	17	8.21	**0.89**	0.20	0.09	0.14	0.13	0.18	0.20	0.02	0.18	3.0	0.60	**0.39**	1.63	8
Santa Rosa Flats	463	4	4	73	191	127	322	17	6.96	**0.76**	0.10	0.08	0.09	0.16	0.41	0.38	0.01	0.08	2.0	0.40	0.24	1.46	9
Tanner Bank	3,112	40	21	126	136	224	233	18	8.22	**0.89**	0.17	0.06	0.12	0.04	0.04	0.06	0.01	0.06	1.0	0.20	0.13	1.20	10
San Nicolas Island	861	11	8	69	128	107	260	16	5.74	**0.62**	0.08	0.07	0.07	0.08	0.15	0.15	0.01	0.09	3.0	0.60	0.35	1.19	11
Mission Beach Reef	361	5	3	205	12	548	18	14	2.03	0.22	0.42	0.00	**0.21**	0.57	0.03	**0.40**	0.01	0.08	3.0	0.60	0.34	1.17	12
43 Fathom Bank	1,323	17	80	113	305	246	870	18	3.24	0.35	0.19	0.22	**0.21**	0.09	0.23	0.21	0.06	0.58	1.0	0.20	0.39	1.16	13
107–118 Bank	617	5	8	37	353	49	624	15	3.85	0.42	0.04	0.16	0.10	0.06	0.57	**0.42**	0.01	0.12	1.0	0.20	0.16	1.10	14
San Miguel Island	431	4	9	57	57	90	143	11	4.76	0.52	0.07	0.04	0.05	0.13	0.13	0.18	0.02	0.20	2.0	0.40	0.30	1.05	15
Cherry Bank	1,220	12	6	140	364	304	1,047	14	4.42	0.48	0.23	0.27	**0.25**	0.11	0.30	0.27	0.00	0.05	0.0	0.00	0.03	1.03	16
North San Clemente Island	161	3	2	49	2	56	3	10	3.03	0.33	0.04	0.00	0.02	0.30	0.01	0.21	0.01	0.12	4.0	0.80	**0.46**	1.02	17
Kidney Bank	772	10	4	52	89	93	113	17	6.65	0.72	0.07	0.03	0.05	0.07	0.12	0.12	0.01	0.05	1.0	0.20	0.13	1.02	18
Cortes Bank	793	9	6	99	43	174	83	15	7.09	0.77	0.13	0.02	0.08	0.12	0.05	0.12	0.01	0.07	0.0	0.00	0.04	1.00	19
Santa Barbara Island	853	7	9	62	100	182	192	14	5.58	0.61	0.14	0.05	0.09	0.07	0.12	0.13	0.01	0.10	1.0	0.20	0.15	0.98	20
W San Miguel Island	225	3	0	1	54	1	95	6	3.38	0.37	0.00	0.02	0.01	0.00	0.24	0.16	0.00	0.00	4.0	0.80	**0.40**	0.94	21
E Santa Catalina Island	188	2	0	5	6	7	6	6	5.44	0.59	0.01	0.00	0.00	0.03	0.03	0.04	0.00	0.00	3.0	0.60	0.30	0.93	22
Central San Clemente	222	3	0	78	9	154	12	11	3.72	0.40	0.12	0.00	0.06	0.35	0.04	0.26	0.00	0.00	2.0	0.40	0.20	0.92	23
Santa Rosa Island	224	3	0	87	18	162	39	11	2.96	0.32	0.12	0.01	0.07	0.39	0.08	0.31	0.00	0.00	2.0	0.40	0.20	0.90	24
Potato Bank	1,069	11	5	15	335	39	585	12	3.35	0.36	0.03	0.15	0.09	0.01	0.31	0.22	0.00	0.04	2.0	0.40	0.22	0.89	25
Cortes Spawning Grd	824	3	8	30	242	38	352	12	4.02	0.44	0.03	0.09	0.06	0.04	0.29	0.22	0.01	0.09	1.0	0.20	0.15	0.86	26
Lausen Knoll	379	3	5	9	39	16	53	10	4.08	0.44	0.01	0.01	0.01	0.02	0.10	0.08	0.01	0.13	2.0	0.40	0.27	0.81	27
57 Fathom Reef	244	2	0	32	42	60	65	16	4.31	0.47	0.05	0.02	0.03	0.13	0.17	0.20	0.00	0.00	1.0	0.20	0.10	0.80	28
Hidden Reef	320	3	6	48	54	166	112	8	3.83	0.42	0.13	0.03	0.08	0.15	0.17	0.21	0.02	0.18	0.0	0.00	0.09	0.80	29
Cortez Ridge	394	5	4	13	92	28	187	8	2.91	0.32	0.02	0.05	0.03	0.03	0.23	0.18	0.01	0.10	2.0	0.40	0.25	0.78	30
117 Seamount	308	2	2	22	131	31	326	13	2.39	0.26	0.02	0.08	0.05	0.07	0.43	0.33	0.01	0.06	1.0	0.20	0.13	0.77	31
Osborne Bank	574	6	4	55	59	144	80	14	3.81	0.41	0.11	0.02	0.07	0.10	0.10	0.13	0.01	0.07	1.0	0.20	0.14	0.75	32

**Notes:**

Richness, abundance, frequency, and fishing intensity (RAFi) index scores for all observed coral and sponge taxa in Southern California dive sites (*n* = 32). Columns are site name (Locality); number of dives with 30 images or more (Full Transects); number of photos deeper than 45 m (in blue); number of photos of gear, sponges, and corals (in yellow); total counts of colonies observed (orange); richness score based on Simpson’s diversity (purple); abundance and frequency of corals and sponges (green); and ranked fishing pressure (0-5) based on map symbol categories from Jenk’s Natural Breaks (in gray). Values in bold indicate the upper quartile scores for each category of interest. The RAFi index (red) indicates combined values for all four parameters with a maximum possible score of 4.00.

* Indicate percentiles for each parameter.

**Table 8:** RAFi for vulnerable taxa.

Vulnerable taxa	Observations		Occurrences			Colonies		Richness			Abundance			Frequency			Fishing Intensity					*RAFi*	
Locality	*n*	Transect count	Gear	Coral occur	Sponge occur	Coral total	Sponge total	Total taxa	Simpson’s 1/D	R score*	Abundance CO	Abundance SP	A score*	Coral ratio	Sponge ratio	F score*	Gear ratio	Gear score	Land	Land score	Fl score*	Sum	Rank
Piggy Bank	736	3	9	425	203	607	284	6	9.2	**1.00**	0.78	0.21	**0.49**	0.58	0.05	**1.00**	0.01	0.12	3.00	0.60	0.36	**2.85**	**1**
The Footprint	2,170	26	34	430	525	779	1,368	9	6.21	0.68	1.00	1.00	**1.00**	0.20	0.09	**0.45**	0.02	0.15	3.00	0.60	0.38	**2.50**	**2**
W Santa Catalina Island	195	2	19	89	0	170	0	6	4.67	0.51	0.22	0.00	0.11	0.46	0.00	**0.73**	0.10	0.94	5.00	1.00	**0.97**	**2.32**	**3**
Farnsworth Bank	643	9	67	142	3	382	4	6	4.05	0.44	0.49	0.00	**0.25**	0.22	0.00	0.36	0.10	1.00	4.00	0.80	**0.90**	**1.95**	**4**
S San Clemente Island	932	11	13	73	56	94	65	10	8.73	**0.95**	0.12	0.05	0.08	0.08	0.03	0.18	0.01	0.13	4.00	0.80	**0.47**	**1.68**	**5**
9-Mile Bank	1,569	15	30	109	124	133	148	6	8.21	**0.89**	0.17	0.11	0.14	0.07	0.03	0.16	0.02	0.18	3.00	0.60	0.39	**1.58**	**6**
109 Seamount	268	1	19	67	62	177	111	5	5.64	0.61	0.23	0.08	**0.15**	0.25	0.03	**0.45**	0.07	0.68	0.00	0.00	0.34	**1.56**	**7**
Del Mar Steeples	202	4	14	56	0	94	0	4	2.22	0.24	0.12	0.00	0.06	0.28	0.00	**0.44**	0.07	0.67	3.00	0.60	**0.64**	1.38	8
43 Fathom Bank	1,323	17	80	82	169	191	623	7	3.24	0.35	0.25	0.46	**0.35**	0.06	0.11	0.28	0.06	0.58	1.00	0.20	0.39	1.37	9
Santa Rosa Flats	463	4	4	9	76	9	115	6	6.96	0.76	0.01	0.08	0.05	0.02	0.09	0.17	0.01	0.08	2.00	0.40	0.24	1.22	10
Tanner Bank	3,112	40	21	57	42	84	101	7	8.22	**0.89**	0.11	0.07	0.09	0.02	0.01	0.05	0.01	0.06	1.00	0.20	0.13	1.16	11
San Nicolas Island	861	11	8	22	35	25	53	7	5.74	0.62	0.03	0.04	0.04	0.03	0.04	0.10	0.01	0.09	3.00	0.60	0.35	1.10	12
North San Clemente Island	161	3	2	27	0	38	1	3	3.03	0.33	0.05	0.00	0.02	0.17	0.00	0.27	0.01	0.12	4.00	0.80	**0.46**	1.08	13
Cortes Bank	793	9	6	42	30	82	54	5	7.09	**0.77**	0.11	0.04	0.07	0.05	0.03	0.14	0.01	0.07	0.00	0.00	0.04	1.02	14
San Miguel Island	431	4	9	29	5	40	7	4	4.76	0.52	0.05	0.01	0.03	0.07	0.01	0.13	0.02	0.20	2.00	0.40	0.30	0.97	15
Santa Rosa Island	224	3	0	53	0	112	0	2	2.96	0.32	0.14	0.00	0.07	0.24	0.00	0.38	0.00	0.00	2.00	0.40	0.20	0.97	16
Mission Beach Reef	361	5	3	72	0	131	0	5	2.03	0.22	0.17	0.00	0.08	0.20	0.00	0.32	0.01	0.08	3.00	0.60	0.34	0.96	17
Kidney Bank	772	10	4	24	41	27	53	7	6.65	0.72	0.03	0.04	0.04	0.03	0.01	0.07	0.01	0.05	1.00	0.20	0.13	0.95	18
Central San Clemente	222	3	0	33	6	66	7	6	3.72	0.40	0.08	0.01	0.04	0.15	0.02	0.27	0.00	0.00	2.00	0.40	0.20	0.92	19
Santa Barbara Island	853	7	9	16	50	24	94	7	5.58	0.61	0.03	0.07	0.05	0.02	0.05	0.10	0.01	0.10	1.00	0.20	0.15	0.91	20
E Santa Catalina Island	188	2	0	0	1	0	1	1	5.44	0.59	0.00	0.00	0.00	0.00	0.00	0.00	0.00	0.00	3.00	0.60	0.30	0.89	21
Cortes Spawning Grd	824	3	8	6	142	9	209	5	4.02	0.44	0.01	0.15	0.08	0.01	0.13	0.22	0.01	0.09	1.00	0.20	0.15	0.89	22
Cherry Bank	1,220	12	6	86	94	204	152	7	4.42	0.48	0.26	0.11	0.19	0.07	0.04	0.17	0.00	0.05	0.00	0.00	0.03	0.86	23
W San Miguel Island	225	3	0	0	15	0	18	2	3.38	0.37	0.00	0.01	0.01	0.00	0.03	0.05	0.00	0.00	4.00	0.80	0.40	0.82	24
107–118 Bank	617	5	8	28	102	35	138	5	3.85	0.42	0.04	0.10	0.07	0.05	0.06	0.17	0.01	0.12	1.00	0.20	0.16	0.82	25
Lasuen Knoll	379	3	5	3	24	3	31	3	4.08	0.44	0.00	0.02	0.01	0.01	0.05	0.09	0.01	0.13	2.00	0.40	0.27	0.81	26
Potato Bank	1,069	11	5	5	124	12	171	5	3.35	0.36	0.02	0.13	0.07	0.00	0.09	0.15	0.00	0.04	2.00	0.40	0.22	0.81	27
57 Fathom Reef	244	2	0	17	14	43	15	6	4.31	0.47	0.06	0.01	0.03	0.07	0.03	0.16	0.00	0.00	1.00	0.20	0.10	0.77	28
Osborne Bank	574	6	4	23	30	27	36	7	3.81	0.41	0.03	0.03	0.03	0.04	0.04	0.12	0.01	0.07	1.00	0.20	0.14	0.70	29
Cortez Ridge	394	5	4	0	29	0	37	2	2.91	0.32	0.00	0.03	0.01	0.00	0.07	0.11	0.01	0.10	2.00	0.40	0.25	0.69	30
Hidden Reef	320	3	6	10	14	10	16	3	3.83	0.42	0.01	0.01	0.01	0.03	0.04	0.12	0.02	0.18	0.00	0.00	0.09	0.64	31
117 Seamount	308	2	2	8	29	9	32	5	2.39	0.26	0.01	0.02	0.02	0.03	0.03	0.09	0.01	0.06	1.00	0.20	0.13	0.50	32

**Notes:**

Richness, abundance, frequency, and fishing intensity (RAFi) index scores for vulnerable taxa in Southern California dive sites (*n* = 32). Columns are site name (Locality); number of dives with 30 images or more (Full Transects); number of photos deeper than 45 m (in blue); number of photos of gear, sponges, and corals (in yellow); total counts of colonies observed (orange); richness score based on Simpson’s diversity (purple); abundance and frequency of corals and sponges (green); and ranked fishing pressure (0–5) based on map symbol categories from Jenk’s Natural Breaks (in gray). The RAFi index (red) indicates combined values for all four parameters with a maximum possible score of 4.00. Values in bold indicate the upper quartile scores for each category of interest.

* Indicate percentiles for each parameter.

**Table 9:** Compared rankings.

Rank	RAFi	Richness-abundance	Richness-frequency	Richness-abundance-frequency
1	Piggy Bank	The Footprint	Piggy Bank	Piggy Bank
2	The Footprint	Piggy Bank	W Catalina Island	The Footprint
3	W Catalina Island	*S San Clemente Island*	The Footprint	W Catalina Island
4	**Farnsworth Bank**	9-Mile Bank	*S San Clemente Island*	*S San Clemente Island*
5	S San Clemente Island	*Tanner Bank*	109 Seamount	109 Seamount
6	9-Mile Bank	*Cortes Bank*	9-Mile Bank	9-Mile Bank
7	109 Seamount	*Santa Rosa Flats*	*Tanner Bank*	Farnsworth Bank
8	**Del Mar Steeples**	Kidney Bank	*Santa Rosa Flats*	*Tanner Bank*
9	43 Fathom Bank	109 Seamount	*Cortes Bank*	*Santa Rosa Flats*
10	Santa Rosa Flats	43 Fathom Bank	Farnsworth Bank	*Cortes Bank*

**Note:**

Table comparing rankings of “top 10” sites for corals and sponges in Southern California, with and without fishing intensity values. The RAFi column indicates ranks based on richness, abundance, and fishing intensity. Other columns show ranks without fishing parameters. **Bold** shows sites that rank higher with fishing, and *italics* show sites that rank higher without fishing.

The “All taxa” method, which included smaller taxa, ranked Piggy Bank, 9-Mile Bank, and South San Clemente Island as highest for species diversity. Piggy Bank, The Footprint, Cherry Bank, and 43-Fathom Bank scored highest for abundance of corals and sponges. Piggy Bank, The Footprint, and Del Mar Steeples scored highest for frequency of corals and sponges. Farnsworth Bank, West Santa Catalina Island, and Del Mar Steeples scored highest for fishing intensity. When the ranking was repeated using only vulnerable taxa, which excluded some of the smaller taxa, the top scores for richness (in order) were Piggy Bank, South San Clemente Island, and 9-Mile Bank; the top scores for abundance were Piggy Bank, The Footprint, and 43-Fathom Bank; the top scores for frequency were Piggy Bank, The Footprint, and Del Mar Steeples; and the top scores for fishing intensity were West Santa Catalina Island, Farnsworth Bank, and Del Mar Steeples.

When summed RAFi scores were tallied for all the larger structure-forming taxa, the top six (upper quartile) ranked were Piggy Bank, The Footprint, West Santa Catalina Island, 9-Mile Bank, 109-Seamount, and South San Clemente Island. Upper quartile ranking for vulnerable taxa were Piggy Bank, West Santa Catalina Island, The Footprint, Farnsworth Bank, South San Clemente Island, and 9-Mile Bank. The ranking results of both exercises were highly correlated (*R*^2^ – 0.988) and not statistically different (*p* = 0.03; *p* < 0.05). Six sites consistently ranked the highest even when fishing intensity values were removed from the total RAFi score.

### Geographic overlap between deep benthic communities and demersal fishing

Corals and sponges were observed in many of the blocks from which demersal fisheries landings were reported, particularly near San Diego and several offshore islands (Figs. 13A and 13B and Figs. S2–S4). The specific fisheries operating in overlapping catch blocks and the types of structure-forming invertebrates occurring in those catch blocks are summarized in Table 10. The highest landings were reported from trap and line fisheries. Trap fisheries took prawns, sablefish, and hagfish, with highest landings at Port Hueneme and San Clemente, the latter of which had many documented species of corals (Osada & Cailliet, 1975). The line fisheries took sablefish, thornyheads, and rockfish, with highest landings at Santa Catalina, Piggy Bank, and San Clemente all with records of corals. Trawl landings were highest at the Port of Long Beach and Port Hueneme, but also reported at San Clemente and throughout the Bight.

**Table 10:** Priority areas by gear.

Region	Block	Priority	Corals Observed	Total Landings	Bottom trawl		Traps		Lines		Nets	
					Landings (lbs.)	Fisheries	Landings (lbs.)	Fisheries	Landings (lbs.)	Fisheries	Landings (lbs.)	Fisheries
Long beach	740	3	No data	174,467	**116,071**	RP, SC, SO	48,195	HF, SA	8,462	SA, RF, TH	1,740	SS, SO
Basin/slope	749	3	No data	76,200	2,068	RP, SO	**73,908**	PR	224	TH, SA, RF	0	–
Piggy Bank	707	2	AT, LO, GO	63,883	1,130	SC	7,877	PR, SA	**54,876**	TH, SA, RF	0	–
Piggy Bank	708	2	AT, LO, GO	32,885	2,489	SC	2,467	SA, PR	**27,726**	TH, SA	204	RF, SS, RP
Port Hueneme	683	3	No data	238,716	20,686	RP, SO	**213,568**	PR, HF	1,224	TH	3,238	SS
San Clemente	829	2	STY, GO	74,300	0	–	30,371	PR	**43,929**	SA, TH, RF	0	–
San Clemente	850	2	STY, GO	21,994	**14,229**	SC	4,546	PR	3,219	SA,TH	0	–
San Clemente	830	3	No data	55,607	0	–	381	PR	**55,226**	SA, TH, RF	0	–
San Clemente	867	1	AT, LO, GO	182,836	0	–	**170,810**	PR	12,006	SA, TH, RF	21	na
San Nicolas	812	4	STY, GO, SPG	3,504	0	–	**2,475**	SA	0	–	1,029	SS
San Nicolas	815	4	LO, SPG	17,067	0	–	**17,067**	PR	0	–	0	–
San Nicolas	813	2	STY, GO, SPG	91,255	1,735	SC	**89,120**	PR	0	–	400	SS
San Nicolas	814	2	GO, SPG	69,518	4,024	SC	**64,237**	PR, SA	0	–	1,257	SS
Santa Catalina	760	2	GO, SPG	62,225	90	SO	**45,195**	PR, HF	16,941	TH, SA, RF	0	–
Santa Catalina	806	3	None found	106,160	0	–	**73,883**	PR	32,277	SA, RF, TH	0	–
Santa Catalina	761	3	No data	55,191	2,513	SC	**50,576**	HF, PR	2,102	TH, RF	0	–
Santa Catalina	807	3	No data	72,284	0	–	8,192	PR, HF	**64,091**	SA, TH	0	–
Santa Catalina	762	1	LO, STY, GO, SPG	331,281	11,472	RP, SO	130,955	PR, HF	**188,828**	SA,TH, RF	26	SS
Santa Rosa	710	4	SPG	14,278	0	–	**10,940**	PR, SA	3,224	TH, SA, RF	114	SS, SO
Santa Rosa	730	4	GO	25,830	0	–	10,439	PR, SA	**15,391**	RF, SA, TH	0	–
Santa Rosa	711	3	No data	103,219	235	SC	**89,923**	PR, SA, RF	12,936	SA, RF, TH	125	RO

**Notes:**

Priority areas for monitoring interactions between deep, demersal fisheries and structure-forming corals and sponges. Total landings (in pounds; all gear types), landings for each gear type, and dominant fisheries contributing to landings in each gear type from 2007 to 2011. Landings values in bold indicate gear types contributing highest landings in each catch block. Priority level is ranked as: (1) high priority (high landings and many corals); (2) medium priority (moderate landings and some corals); (3) medium priority (moderate landings with no ROV images); or (4) low priority (low landings, no evidence of corals or sponges in ROV surveys, or both).

Coral codes are: AT, Antipathes; LO, Lophelia; GO, Gorgonians; STY, Stylaster; and SPG, Sponges. Fisheries abbreviations are: RP, ridgeback prawn; SC, red sea cucumber; SO, sole; HF, hagfish; SA, sablefish; PR, spot prawn; RF, rockfish; TH, thornyhead; and SS, soupfin shark.

## Discussion

### California demersal fisheries and coral observations in recent reports

This study took a similar approach to the estimation of fishing effort off California as Miller et al. (2014) did in the broad assessment of fisheries distribution along the West Coast, but differs in that the work here focused on Southern California’s deep-water landings, and included benthic invertebrates (sea cucumbers, rock prawns) and non-groundfish (hagfish, soupfin shark). This is an important difference because invertebrates represent 31% of recent landings since 2007. Miller et al. (2014) established a good precedent for scientific application of CDFW data, showing that historical landings and catch per area were relatively low in Southern California groundfish fisheries compared to Northern California. This suggests that the Southern Californian seafloor may be generally less impacted and more pristine than seafloor habitats in other regions. The reduced impact would help to maintain diverse and abundant benthic epifauna, such as the deep-sea corals and sponges observed in our study.

All the coral genera reported here were previously known to occur in the SCB, but this report adds new information on their geographic and vertical distribution and helps to prioritize particular localities for management. Two different assemblages of azooxanthellate corals were identified: a “shallow” (50–100 m) and a “deep” (100–400 m) assemblage. The reef-forming hydrocoral *Stylaster californicus* was found to be the predominant hard coral in shallower depths. *Stylaster* appeared to occupy the same vertical niche as four gorgonians, *Leptogorgia chilensis*, *Eugorgia rubens*, *Adelogorgia phyllosclera*, and *Muricea* spp. Slightly deeper waters (>100 m) hosted a different assemblage consisting of *Antipathes dendrochristos*, *Paragorgia* sp., and *Lophelia pertusa* (now *Desmophyllum pertusum*), as well as *Plumarella* sp., and *Acanthogorgia* sp. Overall, the composition of the SCB coral assemblage is diverse, with at least twelve structure-forming deep-sea coral genera. Several observations of gorgonian corals could not yet be identified by photos alone, or may represent undescribed taxa.

An important caveat to consider is that coral genus and sponge types were identified based on the limits of visual (photographic) observations and taxa known to exist in the region based on previous surveys where samples were collected. The ROV surveys employed for this study were originally visual surveys for white abalone and groundfish stock assessments, where no biological samples of corals or sponges were collected to verify identifications. However, data derived from these surveys can be used to pinpoint large aggregations of deep-sea corals at the genus level because the genera have distinct coloration and branching patterns. The information can be used in future studies to guide collections of voucher specimens for morphology, genetics, population connectivity, age, and growth studies. More fieldwork is also required to target areas where corals have not been documented but may be expected to occur.

Further analysis of the image data remains to be done. Sponges were enumerated, but only identified by morphological type. Fishes and other benthic megafauna remain to be enumerated. Coral health and condition, size-class structure, and other aspects may also be ascertained from the substantial collection of images amassed by NOAA’s Southwest Fisheries Science Center. The images are now publicly available through NOAA’s National Database of Deep-Sea Corals and Sponges and may be employed for further analyses (NOAA, 2017).

### Overlap of fisheries and deep-sea corals

Three types of spatial overlap were proposed as a framework for understanding co-occurrences of commercial fisheries with corals and sponges in the SCB: co-occurrences (in the same frame) from images of the seafloor, depth range overlap, and geographic overlap. The co-occurrence of corals, sponges, and fishing debris in ROV photos was observed directly and quantified by study site. Depth range overlap was illustrated through an analysis of depth distribution of corals, sponges, and fishing debris observed in benthic ROV survey data. Broad geographic overlap was also illustrated empirically by overlaying coral and sponge occurrences with fisheries landings effort. Depth and geographic area overlaps have a coarse spatial scale. Many seabed types can occur in a single catch block. Different assemblages can be expected to occur in each seabed (or community) type. The highest likelihood of interactions would be expected in rocky habitats, when the preferred habitat of commercially fished species (e.g., rockfish, spot prawn) overlaps with the preferred habitat of corals and sponges.

Future studies should seek to access or generate fine-scale (1–5 m resolution) benthic maps that delineate soft-bottom from hard-bottom in order to avoid accidental incursions into hard-bottom habitats by fisheries targeting soft-bottom areas. It would also be helpful to survey fishermen to ask about the habitat preferences of their target species, and whether fishing efforts targeting certain seabed types are indeed restricted to those types.

From this analysis, it appears that depth range overlap does exist between bottom-contact fisheries and corals and sponges throughout the SCB. The majority of fishing debris in this study was observed in the 50–250 m depth range. This is consistent with the depth range of the largest demersal fisheries in the SCB in terms of total landings, including: set line fisheries for sablefish and shortspine thornyhead, bottom trawls for ridgeback prawn, and traps for hagfish and spot prawn. The 50–250 m depth range is also consistent with the depth range of several species of corals, including *Stylaster californicus*, *Lophelia pertusa*, *Antipathes dendrochristos*, *Leptogorgia chilensis*, *Eugorgia rubens*, *Adelogorgia* sp., *Plumarella* sp., and *Acanthogorgia* sp. The bubblegum coral *Paragorgia* is slightly deeper than this range, but there are reports that commercial and recreational rockfish fisheries have been moving into deeper waters (Leet, 2001; Miller et al., 2014) where *Paragorgia* and other fragile sea fans are expected to occur.

In terms of geographic overlap, these results show that mobile gear has a relatively small footprint in the SCB compared to fixed gear, and there is considerable overlap between fisheries landings and the occurrence of corals and sponges for both mobile (trawl) and fixed gear. Fixed gear is more often used in complex, rocky areas whereas trawl gear is more often used in broad flat areas. Complex rocky areas are a hazard for trawl gear. It is evident from this study that trawling has occurred since 2007 in some of the same catch blocks as corals and sponges; however, in situ photo observations employed for this study were focused primarily on offshore habitats. So, the true extent of the overlap in the Santa Barbara Channel is not fully realized. An aggregated dataset combining multiple resources (e.g., observations from towed cameras and submersibles) is necessary to deduce the full extent of geographic overlap in nearshore habitats in the northern SCB where most trawl landings originate. NOAA’s Deep-Sea Coral Research Technology Program currently assembles this type of public resource (NOAA, 2017).

A large portion of fixed gear landings occur offshore compared to mobile gear, and a large number of in situ observations of corals and sponges were also offshore. Our analysis of co-occurrence from ROV photos showed that lines and rope were the most commonly observed fishing debris based on the frequency of occurrence and the frequency of interactions with corals and sponges. Bottom longline fisheries are an emerging concern for deep-sea coral management because of coral bycatch in these fisheries (Sampaio et al., 2012). Line fisheries have traditionally not been a concern because “area swept” is small compared to mobile gear. However, entanglements do occur, and the total landings by those fisheries are high, so cumulative impacts may bear investigation (Rooper et al., 2017). Traps also have the potential to damage corals and sponges on deployment and recovery, especially when strung together.

### Priority sites for conservation- and management-based research and monitoring

A qualitative analysis was used to prioritize catch blocks in terms of potential spatial overlap with observations of deep, structure-forming corals and sponges based upon (1) the abundance and richness of corals and sponges, (2) the relative magnitude of landings within each gear type, and (3) the number of different gear types used in each block. Three priority types emerged from this analysis: high priority (high landings and many corals); medium priority (moderate landings and some corals or moderate landings with no ROV images); and low priority (low landings, no evidence of corals or sponges in ROV surveys, or both) (Fig. S1).

Highest priority recommendations for conservation-focused research of deep-water corals include sites off San Clemente Island and Santa Catalina Island, particularly in relation to San Clemente Island’s fishery for red sea cucumber in block 850; trap fishery for spot prawn in blocks 867 and 829; and set line fisheries for sablefish, rockfishes, and thornyheads in blocks 829 and 830. Block 830 has some fishing but no survey data. Waters within fishable depths around Santa Catalina are closed to bottom trawling. Santa Catalina Island’s set line and trap fisheries in block 762 (high landings, many corals) and blocks 761 and 807 (no survey data) should also be a focus for future research. In addition, block 683 near Port Hueneme has the highest reported landings of demersal species, but no ROV survey data.

Medium priority recommendations for conservation-focused research of deep corals include catch blocks 813 and 814 near San Nicolas; catch blocks 707, 708, and 710 in Channel Islands National Marine Sanctuary (CINMS); and the adjacent catch block 730. San Nicolas reports moderate to low landings in trap fishery for spot prawn, trawl fishery for red sea cucumber, and set net fishery for soupfin sharks. CINMS catch blocks 707 and 708 have moderate landings in the set line fisheries for sablefish, rockfish, and thornyhead. Piggy Bank and The Footprint are mound and ridge features encompassed by these catch blocks. The features have high abundance of corals and sponges. Piggy Bank was the subject of recent research surveys sponsored by NOAA’s Deep-Sea Coral Research and Technology Program (Yoklavich et al., 2011). The Footprint was the subject of benthic surveys by *Delta* submersible (Bright, 2007).

Further west in CINMS, near Santa Rosa, catch blocks 710 and 730 have low landings, but some occurrences of sponges and gorgonians that indicate suitable habitat. Other regions where fishing is occurring but no survey data is available include block 740 off Long Beach, block 711 off Santa Rosa, and block 807 off Santa Catalina (see Table 10, Rank 3).

### Observations of debris in other areas

Unfortunately, anthropogenic marine debris is relatively common along the California coast (Watters et al., 2010) where deep-water corals and sponges occur. In that study, the relative abundance of debris was much higher in central California compared to the SCB. In central California, most observations of debris (over 90%) were of recreational fishing gear (e.g., monofilament fishing line) in rocky habitats compared to the SCB where commercial fishing gear (e.g., longlines, nets, traps, and empty bait cans) and maritime debris (e.g., beverage containers and construction materials) comprised a large portion of observations.

There was little indication in Watters et al. (2010) that the observed debris had caused disturbance to the seabed habitats and/or benthic megafauna present. However, in this study there were 294 examples of co-occurrence with corals and sponges. Co-occurrence was defined as presence of fishing gear and corals or sponges in the same photo. Co-occurrence does not necessarily mean disturbance, but shows derelict gear in close proximity, or in physical contact with benthic fauna observed. Lines and rope were the most common type of debris, constituting nearly 71% of gear co-occurring with structure-forming corals and sponges in this study.

### Site-based prioritization using RAFi index

California Department of Fish and Wildlife landings are reported by catch block, not geographic location, so although fishing may clearly be taking place, fishing may not be taking place in the same habitat type as corals and sponges. For example, rocky substrates favored by spot prawns and certain rockfish are similar to coral and sponge habitats observed. Other target fisheries like sea cucumber and ridgeback prawn favor soft sediments where corals and sponges are less common. Overlap of commercial landing records by catch block with coral and sponge observations can provide some means for the development of effective, priority-based management efforts. However, when determining very refined boundaries for potential HAPCs, catch block-based managements will not distinguish seafloor features or substrate variation within a grid cell. Any evidence from direct observations of fishing gear and coral interactions are also not taken into account using a catch-block approach.

In addition to spatial overlap, the RAFi index incorporated data from frequency of fishing debris observations, as well as diversity of sessile epifauna, to achieve an array of quantitative criteria for conservation and management-based research and monitoring prioritization. Our top results from the RAFi index score (Tables 7 and 8) coincide with priority blocks for Piggy Bank, The Footprint, Western Catalina Island, and Southern San Clemente Island (Table 10 and Fig. S1). When fishing is excluded, the study sites at South San Clemente, Santa Rosa Flats, 9-Mile Bank, Tanner Banks, Cortes Bank, and West Catalina are all up-ranked priorities in the top 10, while and Del Mar Steeples and Farnsworth Bank drop in rank (Table 9). The RAFi index is therefore flexible and useful for a variety of prioritization approaches.

## Conclusions

In conclusion, this study shows that deep-water corals and sponges are broadly distributed offshore and throughout the SCB, from Point Conception in the north to San Diego in the south. The general depth ranges of corals observed in this study (45–500 m) are consistent with the depth ranges of commercial fishing debris and the depth ranges of bottom-contact fisheries as reported by the state fisheries agency.

As reported in Rooper et al. (2017), the spatial extent of fixed gear fisheries in Southern California is found to be larger than the extent of trawl fisheries, which is perhaps of some concern for corals and sponges because most co-occurrences were from lines. More information is needed on the distribution of corals and sponges precisely where fixed gear and trawl fisheries operate in the SCB. Any rocky outcrop offers potential habitat to deep-sea corals and sponges (Hardin et al., 1994). There is also a need for more information on the absence of corals and sponges where bottom-contact fisheries occur.

This study assigned priority ranks based on a conservation priority index called “RAFi” scores. An index such as RAFi can be a useful decision support tool for marine managers to help focus habitat conservation efforts. In this case, the ranking exercise indicated that an existing network of State marine reserves, National marine sanctuaries, EFH, and Rockfish conservation areas (RCAs) (Pacific Fishery Management Council, 2006) already encompasses most of the highly-ranked localities, and this limits impacts from bottom-trawling. Based upon data examined, the Channel Islands region, San Clemente Island, Santa Catalina Island, and sites offshore like Seamount 109 should be a high priority for research and management. In these places, diversity and abundance of deep-sea corals and sponges is high, and the bottom fishing restrictions in place in these areas are impermanent, and routinely under review by the regional Fisheries Management Councils. The benthic communities at Cortes Bank, Tanner Bank, and Cherry Bank had moderate diversity, high abundance and low fishing pressure, so these ranked as intermediate priorities, but they gain importance if fishing intensity is not included. The Cowcod Conservation Area encompasses these outer banks.

The largest coastal protected area in Southern California at the time of this writing is the RCA, which encompasses many highly-ranked localities. The extent of the RCA fluctuates seasonally, and the regulation is impermanent. Wherever RCA is in place, de facto protections from bottom trawling may be expected to provide benefits to deep-sea corals and sponges, as well as rockfish. A new area called the SCB EFH conservation area is now recommended by the Pacific Fishery Management Council (2018) to permanently restrict trawl gear where most deep-water coral and sponges occur in the SCB. The proposal will not restrict bottom-contact gear such as longlines, pots, and traps, which are most likely to be found in these hard-bottom ecosystems. Ongoing dialogue with fishermen, improved habitat maps, and continued benthic surveys are recommended to determine where fisheries interactions occur and whether deep-sea corals and sponges are adversely affected.

## Supplemental Information

10.7717/peerj.5697/supp-1 Supplemental Information 1 Table S1. Scientific names. Scientific names for Southern California demersal, deep-water (>50 m) fisheries landings categories. *cf. *Pandalus jordani*.

10.7717/peerj.5697/supp-2 Supplemental Information 2 Table S2. Raw counts of dives and observations for major coral taxa. Counts of dives per site, number of photos deeper than 45 m, images of corals, and counts per major taxa. *Lophelia pertus*a (LO), *Desmophyllum* sp. (DES), Coenocyathus (CEN), *Dendrophyllia* sp. (DEN), and* Stylaster californicus* (STY) . Columns in orange show the number of colonies for *Acanthogorgia* (ACN), *Adelogorgia* (ADL), *Antipathes dendrochristos* (ANT), *Eugorgia* (EU), *Leptogorgia* (LEP), *Muricea* (MUR), *Paragorgia* (PAR)), *Parastenella* (PRS), *Plumarella* (PLU), *Swiftia* (SWF), unidentified Plexauridae (PLX), and unidentified gorgonians (GOR).

10.7717/peerj.5697/supp-3 Supplemental Information 3 Table S3. Raw counts of dives per site and sponge morph types. Counts of dives per site, number of photos deeper than 45 m, and sponge colonies by morphologic type. Barrel sponges, Shelf sponges, Vase sponges, Branching sponges, Foliose sponges, Globular sponges, and other morphotypes combined.

10.7717/peerj.5697/supp-4 Supplemental Information 4 Fig. S1. Catch blocks and priorities. Catch blocks within the Southern California Bight as determined by California Department of Fish and Wildlife (CDFW, formerly CDFG). Priority areas for research and management relative to catch blocks, protected areas, and landing ports.

10.7717/peerj.5697/supp-5 Supplemental Information 5 Fig. S2. Reported landings commercial fixed gear 2007–2011. Reported landings for commercial fixed gear by catch block from 2007–2011 in the Southern California Bight. Observations of structure-forming gorgonians are also shown, and color-coded by genus.

10.7717/peerj.5697/supp-6 Supplemental Information 6 Fig. S3. Reported trap landings 2007–2011. Reported landings for trap gear by catch block from 2007–2011 in the Southern California Bight. Observations of structure-forming sponges (>10 cm, referred to in the report as ‘3D sponges’) are also shown.

10.7717/peerj.5697/supp-7 Supplemental Information 7 Fig. S4. Reported landings bottom long-lines 2007–2011. Reported landings for bottom set line and long-line by catch block from 2007–2011 in the Southern California Bight. Observations of Antipathes black corals (presumably Antipathes dendrochristos) are also shown.

10.7717/peerj.5697/supp-8 Supplemental Information 8 Fig. S5. Reported landings bottom nets 2007–2011. Reported landings for bottom set nets by catch block from 2007–2011 in the Southern California Bight. Observations of fishing debris (specifically nets, lines, and ropes).

## Additional Information and Declarations

### Data Availability

The following information was supplied regarding data availability: All raw data is publicly available via the National Database of Deep Sea Corals: https://deepseacoraldata.noaa.gov/website/AGSViewers/DeepSeaCorals/mapSites.htm. Fishery landings raw data is publicly available through the California Department of Fish and Wildlife: https://www.wildlife.ca.gov/Fishing/Commercial/Landings. Data relating to Marine protected Areas and fishing block zones: https://www.wildlife.ca.gov/Fishing/Commercial#310591030-selected-fishery-regulation-information.
