# Supplementary material for: Distribution of deep-water corals, sponges, and demersal fisheries landings in Southern California, USA: implications for conservation priorities
Source: PeerJ. 2018 Oct 10;6:e5697. doi: 10.7717/peerj.5697 (PMC6186160; doi:10.7717/peerj.5697)
Supplement: Supplemental Information 1 — Scientific names for Southern California demersal, deep-water (>50 m) fisheries landings categories. *cf. Pandalus jordani. [file peerj-06-5697-s001.pdf]

Species	Scientific name
Hagfishes	Myxinidae
Lingcod	*Ophiodon elongatus*
Prawn, ridgeback	*Sicyonia ingentis*
Prawn, spot	*Pandalus platyceros*
Rockfish, aurora	*Sebastes aurora*
Rockfish, bank	*Sebastes rufus*
Rockfish, black	*Sebastes melanops*
Rockfish, blackgill	*Sebastes melanostomus*
Rockfish, bocaccio	*Sebastes paucispinis*
Rockfish, bronzespotted	*Sebastes gilli*
Rockfish, brown	*Sebastes auriculatus*
Rockfish, canary	*Sebastes pinniger*
Rockfish, chameleon	*Sebastes phillipsi*
Rockfish, chilipepper	*Sebastes goodei*
Rockfish, China	*Sebastes nebulosus*
Rockfish, copper	*Sebastes caurinus*
Rockfish, copper (whitebelly)	*Sebastes caurinus*
Rockfish, cowcod	*Sebastes levis*
Rockfish, darkblotched	*Sebastes crameri*
Rockfish, flag	*Sebastes rubrivinctus*
Rockfish, greenblotched	*Sebastes rosenblatti*
Rockfish, greenspotted	*Sebastes chlorostictus*
Rockfish, greenstriped	*Sebastes elongatus*
Rockfish, group bocaccio/chili	*Sebastes paucispinis/goodei*
Rockfish, group bolina	*Sebastes auriculatus*
Rockfish, group deepwater reds	*Sebastes* spp.
Rockfish, group red	*Sebastes* spp.
Rockfish, group rosefish	*Sebastes* spp.
Rockfish, group shelf	*Sebastes* spp.
Rockfish, group slope	*Sebastes* spp.
Rockfish, group small	*Sebastes* spp.
Rockfish, Mexican	*Sebastes macdonaldi*
Rockfish, olive	*Sebastes serranoides*
Rockfish, Pacific ocean perch	*Sebastes alutus*
Rockfish, pink	*Sebastes eos*
Rockfish, pinkrose	*Sebastes simulator*
Rockfish, redbanded	*Sebastes babcocki*
Rockfish, rosethorn	*Sebastes helvomaculatus*
Rockfish, rosy	*Sebastes rosaceus*
Rockfish, speckled	*Sebastes ovalis*
Rockfish, splitnose	*Sebastes diploproa*
Rockfish, squarespot	*Sebastes hopkinsi*
Rockfish, starry	*Sebastes constellatus*
Rockfish, stripetail	*Sebastes saxicola*
Rockfish, swordspine	*Sebastes ensifer*
Rockfish, unspecified	Sebastes spp.
Rockfish, vermilion	*Sebastes miniatus*
Rockfish, widow	*Sebastes entomelas*
Rockfish, yelloweye	*Sebastes ruberrimus*
Rockfish, yellowtail	*Sebastes flavidus*
Sablefish	*Anoplopoma fimbria*
Sea cucumber, giant red	*Parastichopus californicus*
Sea cucumber, unspecified	Holothuroidea
Shark, soupfin	*Galeorhinus galeus*
Shark, spiny dogfish	*Squalus acanthias*
Shrimp, ocean pink	*Pandalus* spp.*
Sole, butter	*Iopsetta isolepis*
Sole, Dover	*Microstomus pacificus*
Sole, English	*Parophrys vetulus*
Sole, fantail	*Xystreurys liolepis*
Sole, petrale	*Eopsetta jordani*
Sole, rex	*Glyptocephalus zachirus*
Sole, rock	*Lepidopsetta bilineata*
Sole, sand	*Psettichthys melanostictus*
Sole, tongue	Cynoglossidae
Sole, unspecified	Soleidae
Thornyhead, longspine	*Sebastolobus altivelis*
Thornyhead, shortspine	*Sebastolobus alascanus*
Thornyheads	*Sebastolobus* spp.
Trawled fish for animal food	*Sebastes* spp., mostly small
