# Supplementary material for: Distribution of deep-water corals, sponges, and demersal fisheries landings in Southern California, USA: implications for conservation priorities
Source: PeerJ. 2018 Oct 10;6:e5697. doi: 10.7717/peerj.5697 (PMC6186160; doi:10.7717/peerj.5697)
Supplement: Supplemental Information 2 — Counts of dives per site, number of photos deeper than 45 m, images of corals, and counts per major taxa. Lophelia pertusa (LO), Desmophyllum sp. (DES), Coenocyathus (CEN), Dendrophyllia sp. (DEN), and Stylaster californicus (STY) . Columns in orange show the number of colonies for Acanthogorgia (ACN), Adelogorgia (ADL), Antipathes dendrochristos (ANT), Eugorgia (EU), Leptogorgia (LEP), Muricea (MUR), Paragorgia (PAR)), Parastenella (PRS), Plumarella (PLU), Swiftia (SWF), unidentified Plexauridae (PLX), and unidentified gorgonians (GOR). [file peerj-06-5697-s002.pdf]

		Number of Images				Number of Colonies																	
BANK	Total Dives	Photos 45m+	Gear	Coral	Sponge	LO	DES	CEN	DEN	STY	ACN	ADL	ANT	EU	LEP	MUR	PAR	PRS	PLU	SWI	PLX	GOR	Total Coral
Piggy Bank	6	736	9	534	625	129	326	0	0	0	0	0	97	0	0	0	55	0	48	35	4	1	695
The Footprint	31	2033	31	674	1421	65	97	15	0	0	399	166	148	7	1	0	53	0	3	114	32	182	1282
Farnsworth Bank	10	565	67	208	16	0	0	37	1	266	0	108	0	44	69	2	0	0	0	0	0	12	539
109 Seamount	1	268	19	70	167	124	51	0	0	0	0	0	2	0	0	0	0	0	0	1	1	1	180
Santa Catalina Island	6	369	19	169	9	25	3	48	24	0	0	126	0	105	3	10	0	2	0	0	0	17	363
Del Mar Steeples	3	202	15	171	13	8	0	16	0	0	0	319	0	66	19	1	0	0	0	0	0	39	468
Mission Beach Reef	5	345	3	194	12	4	6	7	5	0	0	359	0	106	10	0	0	0	0	4	1	28	530
San Clemente Island	30	1697	16	245	233	16	59	32	3	0	5	78	7	105	36	2	10	3	8	5	0	15	384
43 Fathom Bank	22	1057	80	102	268	26	106	27	53	0	0	1	3	0	0	0	3	0	0	3	0	3	225
Cherry Bank	13	1206	6	141	360	115	13	89	0	73	2	0	0	0	0	0	1	0	11	0	0	0	304
San Miguel Island	17	809	13	70	148	0	0	47	0	3	0	0	0	34	4	0	0	0	0	5	1	22	116
San Nicolas Island	13	990	8	68	154	0	0	49	2	7	0	1	0	12	2	1	0	0	0	0	0	31	105
9 Mile Bank	15	1567	30	180	278	3	105	0	24	0	0	0	0	0	0	0	1	2	65	25	0	6	231
Santa Rosa Flats	5	377	4	54	178	0	0	0	0	0	0	0	4	0	0	0	2	0	82	3	3	2	96
107-118 Bank	5	617	8	47	353	10	11	0	0	0	0	0	2	0	0	0	12	0	0	7	2	1	45
Santa Rosa Island	4	300	0	90	31	0	0	13	0	0	0	40	0	113	2	0	0	0	0	0	1	2	171
Cortes Spawning Gr.	6	824	8	30	242	3	5	0	0	0	0	0	1	0	0	0	0	0	27	0	1	1	38
Tanner Bank	80	2542	21	117	114	11	16	3	0	11	0	2	0	17	29	0	0	0	107	0	0	16	212
Santa Cruz Island	2	79	0	59	2	0	0	5	0	0	0	384	0	6	0	0	0	0	0	0	0	3	398
Potato Bank	13	1047	5	16	332	9	2	24	0	0	0	0	0	0	0	0	1	0	0	3	0	0	39
Santa Barbara Island	7	852	9	49	99	12	0	32	5	0	0	0	4	2	1	0	0	0	3	0	0	110	169
117 Seamount	2	308	2	27	131	0	0	0	0	0	1	0	2	0	0	0	6	0	0	12	5	0	26
60 Mile Bank	5	572	7	14	112	1	1	16	1	0	0	0	0	0	0	0	0	0	0	0	0	0	19
Cortes Bank	11	757	6	90	40	0	0	17	0	15	0	0	0	50	17	0	0	0	47	0	1	13	160
Kidney Bank	7	592	3	29	78	1	17	0	0	0	2	0	1	0	0	0	4	0	0	3	0	1	29
Lasuen Knoll	3	379	5	5	39	0	3	0	0	0	0	0	0	0	0	0	0	0	0	1	0	1	5
Hidden Reef	3	132	3	24	21	1	0	23	0	0	0	0	4	0	0	0	0	0	0	0	0	55	83
Osborne Bank	6	574	4	51	59	5	0	17	1	0	0	90	7	13	0	0	1	0	1	0	0	3	138
Total	331	21796	401	3528	5535	568	821	517	119	375	409	1674	282	680	193	16	149	7	402	221	52	565	7050
