# Supplementary material for: Distribution of deep-water corals, sponges, and demersal fisheries landings in Southern California, USA: implications for conservation priorities
Source: PeerJ. 2018 Oct 10;6:e5697. doi: 10.7717/peerj.5697 (PMC6186160; doi:10.7717/peerj.5697)
Supplement: Supplemental Information 3 — Counts of dives per site, number of photos deeper than 45 m, and sponge colonies by morphologic type. Barrel sponges, Shelf sponges, Vase sponges, Branching sponges, Foliose sponges, Globular sponges, and other morphotypes combined. [file peerj-06-5697-s003.pdf]

		Number of Images				Number of Colonies							
BANK	Total Dives	Photos 45m+	Gear	Coral	Sponge	BARREL	SHELF	VASE	BRANCH	FOLIOSE	GLOBULAR	OTHER	Total Sponge
Piggy Bank	6	736	9	**534**	**625**	45	224	**239**	32	308	**210**	1	**1059**
The Footprint	**31**	**2033**	31	**674**	**1421**	**304**	**392**	**1053**	**156**	**1672**	**323**	**16**	**3916**
Farnsworth Bank	10	565	**67**	208	16	3	0	0	7	5	4	3	22
109 Seamount	1	268	19	70	167	13	99	98	3	61	38	1	313
Santa Catalina Island	6	369	19	169	9	0	2	1	0	8	2	0	13
Del Mar Steeples	3	202	15	171	13	0	1	0	0	21	3	1	26
Mission Beach Reef	5	345	3	194	12	0	0	0	0	16	2	0	18
San Clemente Island	30	1697	16	245	233	52	88	41	5	145	17	14	362
43 Fathom Bank	22	1057	**80**	102	268	**570**	141	20	0	68	3	14	816
Cherry Bank	13	1206	6	141	360	61	**402**	91	7	**464**	14	4	1043
San Miguel Island	17	809	13	70	148	40	161	9	**35**	63	14	0	322
San Nicolas Island	13	990	8	68	154	57	111	4	28	102	5	2	309
9 Mile Bank	15	1567	30	180	278	57	56	91	2	106	15	**24**	351
Santa Rosa Flats	5	377	4	54	178	61	83	53	2	65	18	1	283
107-118 Bank	5	617	8	47	353	45	98	92	1	283	100	4	623
Santa Rosa Island	4	300	0	90	31	0	9	1	19	40	9	0	78
Cortes Spawning Gr.	6	824	8	30	242	172	75	37	0	49	7	12	352
Tanner Bank	**80**	**2542**	21	117	114	68	51	7	8	22	39	0	195
Santa Cruz Island	2	79	0	59	2	0	0	1	0	0	1	0	2
Potato Bank	13	1047	5	16	332	135	291	35	6	85	28	1	581
Santa Barbara Island	7	852	9	49	99	69	55	25	0	40	2	0	191
117 Seamount	2	308	2	27	131	10	22	22	2	215	53	2	326
60 Mile Bank	5	572	7	14	112	138	14	18	1	39	7	0	217
Cortes Bank	11	757	6	90	40	43	4	4	1	21	3	0	76
Kidney Bank	7	592	3	29	78	13	17	34	1	18	16	3	102
Lasuen Knoll	3	379	5	5	39	25	15	6	0	7	0	0	53
Hidden Reef	3	132	3	24	21	4	37	0	0	5	4	0	50
Osborne Bank	6	574	4	51	59	25	21	11	0	23	0	0	80
**Total**	**331**	**21796**	**401**	**3528**	**5535**	**2010**	**2469**	**1993**	**316**	**3951**	**937**	**103**	**11779**
