# Supplementary figures and images for: Distribution of deep-water corals, sponges, and demersal fisheries landings in Southern California, USA: implications for conservation priorities
Source: PeerJ. 2018 Oct 10;6:e5697. doi: 10.7717/peerj.5697 (PMC6186160; doi:10.7717/peerj.5697)

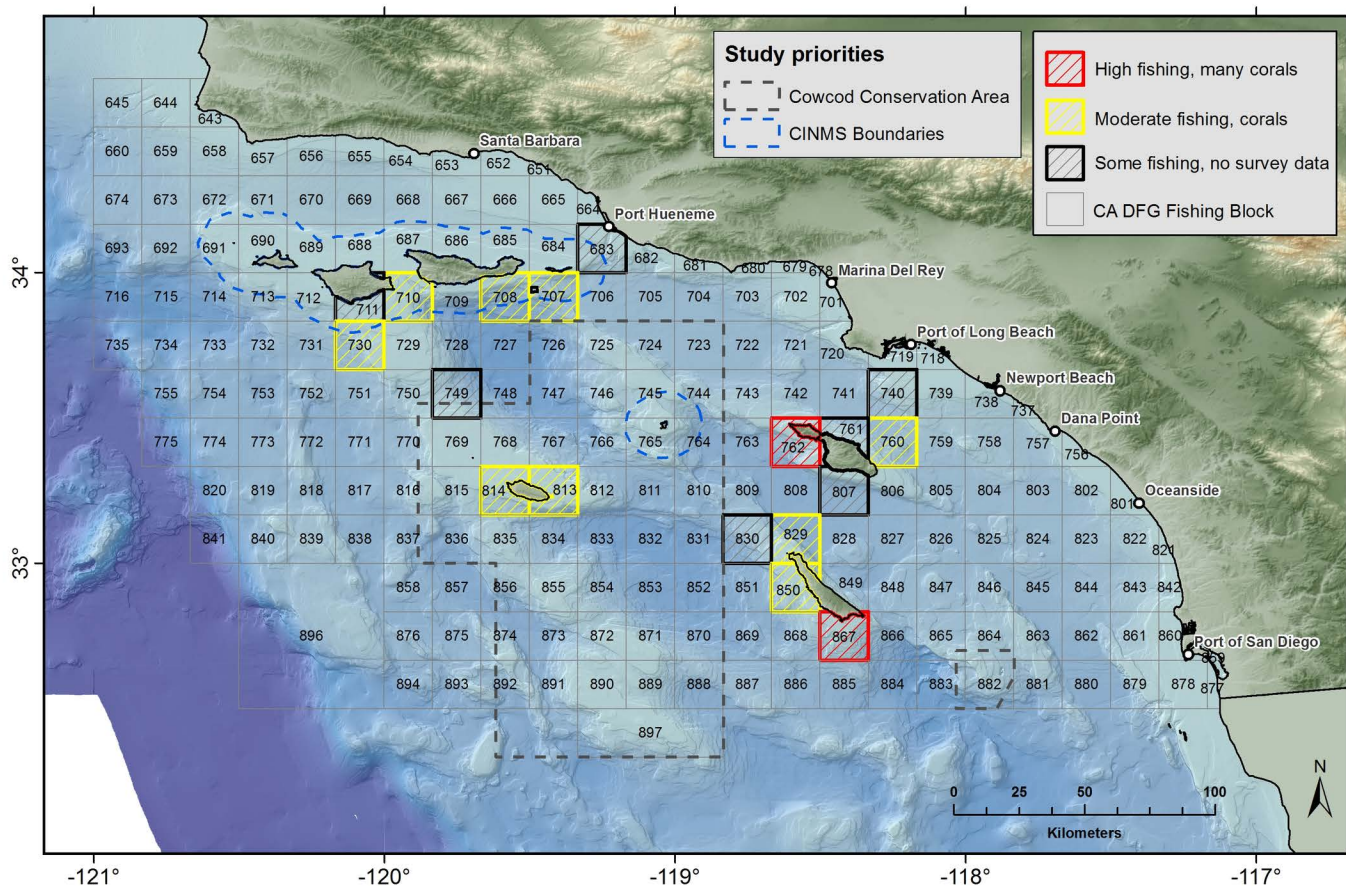

Supplement: Supplemental Information 4 — Catch blocks within the Southern California Bight as determined by California Department of Fish and Wildlife (CDFW, formerly CDFG). Priority areas for research and management relative to catch blocks, protected areas, and landing ports. [file peerj-06-5697-s004.pdf]

34°  
33°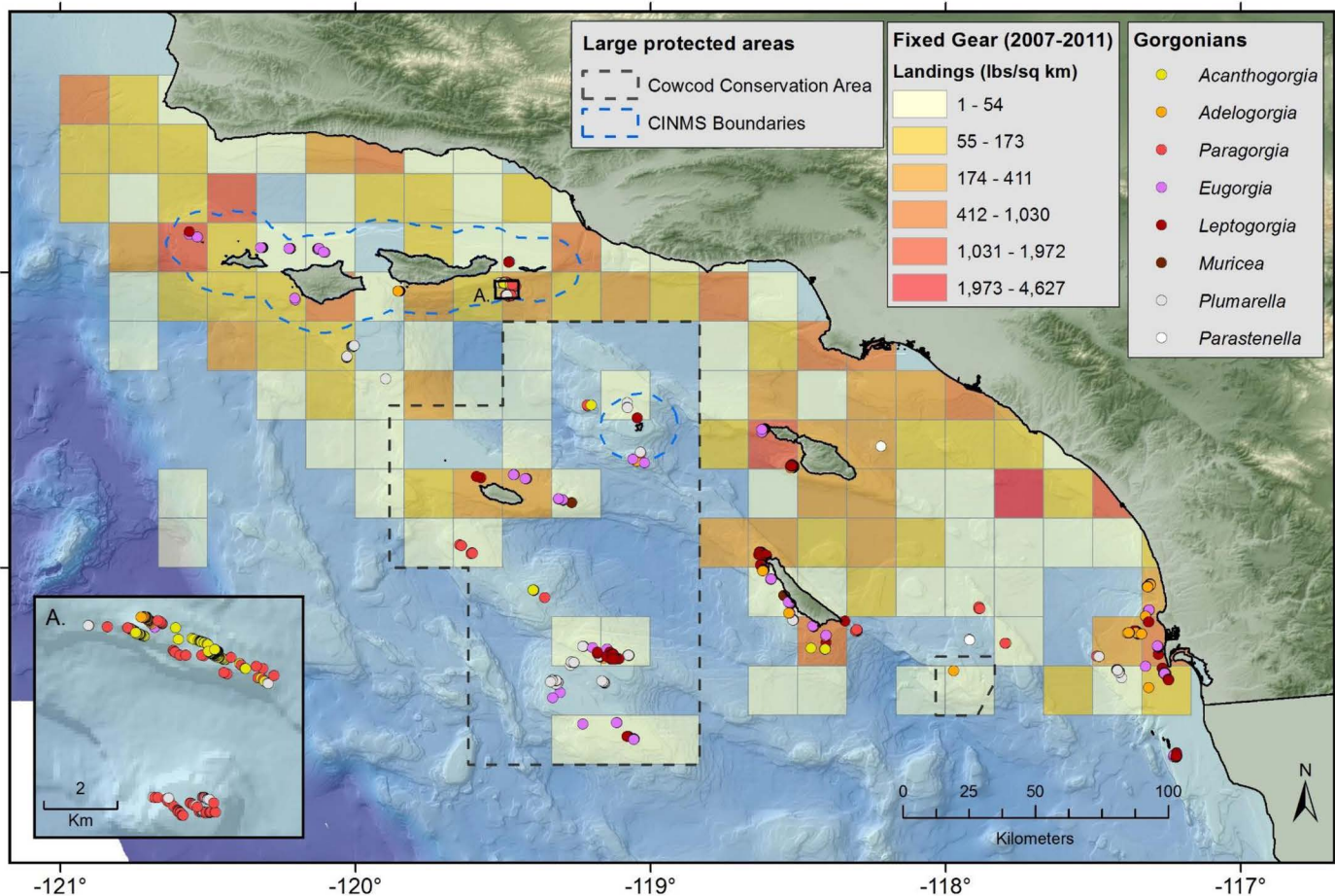

Supplement: Supplemental Information 5 — Reported landings for commercial fixed gear by catch block from 2007–2011 in the Southern California Bight. Observations of structure-forming gorgonians are also shown, and color-coded by genus. [file peerj-06-5697-s005.pdf]

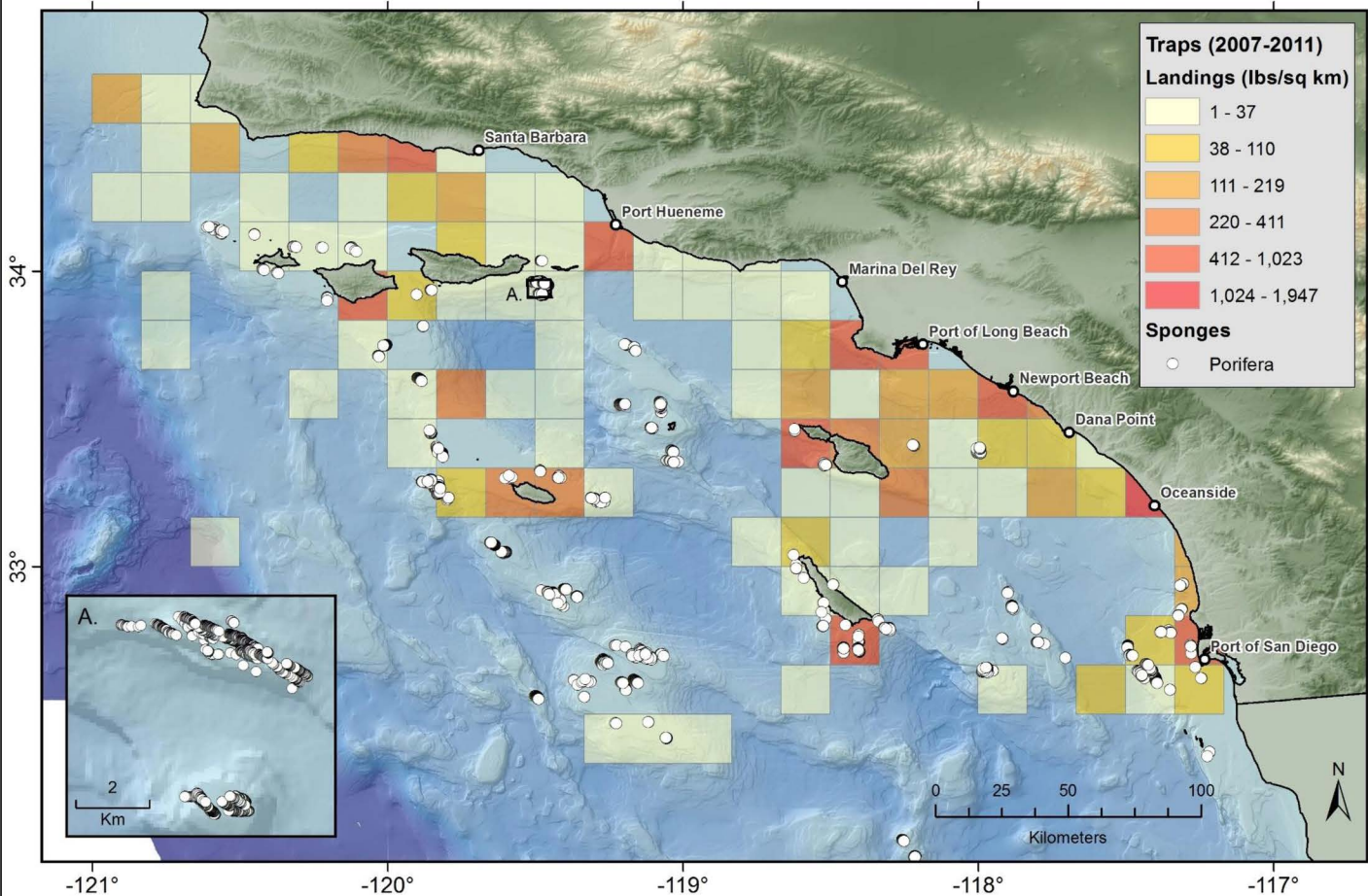

Supplement: Supplemental Information 6 — Reported landings for trap gear by catch block from 2007–2011 in the Southern California Bight. Observations of structure-forming sponges (>10 cm, referred to in the report as ‘3D sponges’) are also shown. [file peerj-06-5697-s006.pdf]

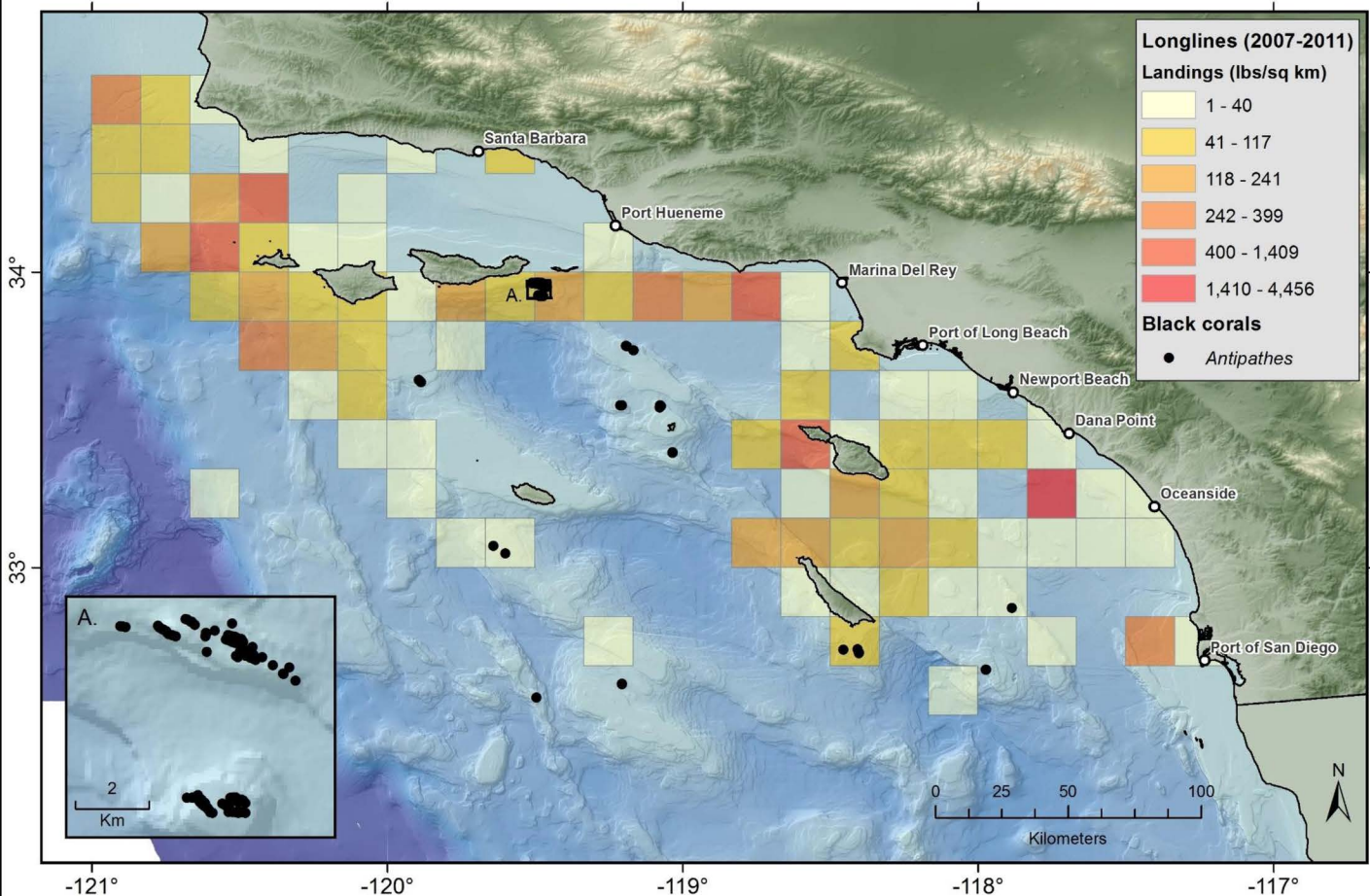

Supplement: Supplemental Information 7 — Reported landings for bottom set line and long-line by catch block from 2007–2011 in the Southern California Bight. Observations of Antipathes black corals (presumably Antipathes dendrochristos) are also shown. [file peerj-06-5697-s007.pdf]

34°

33°

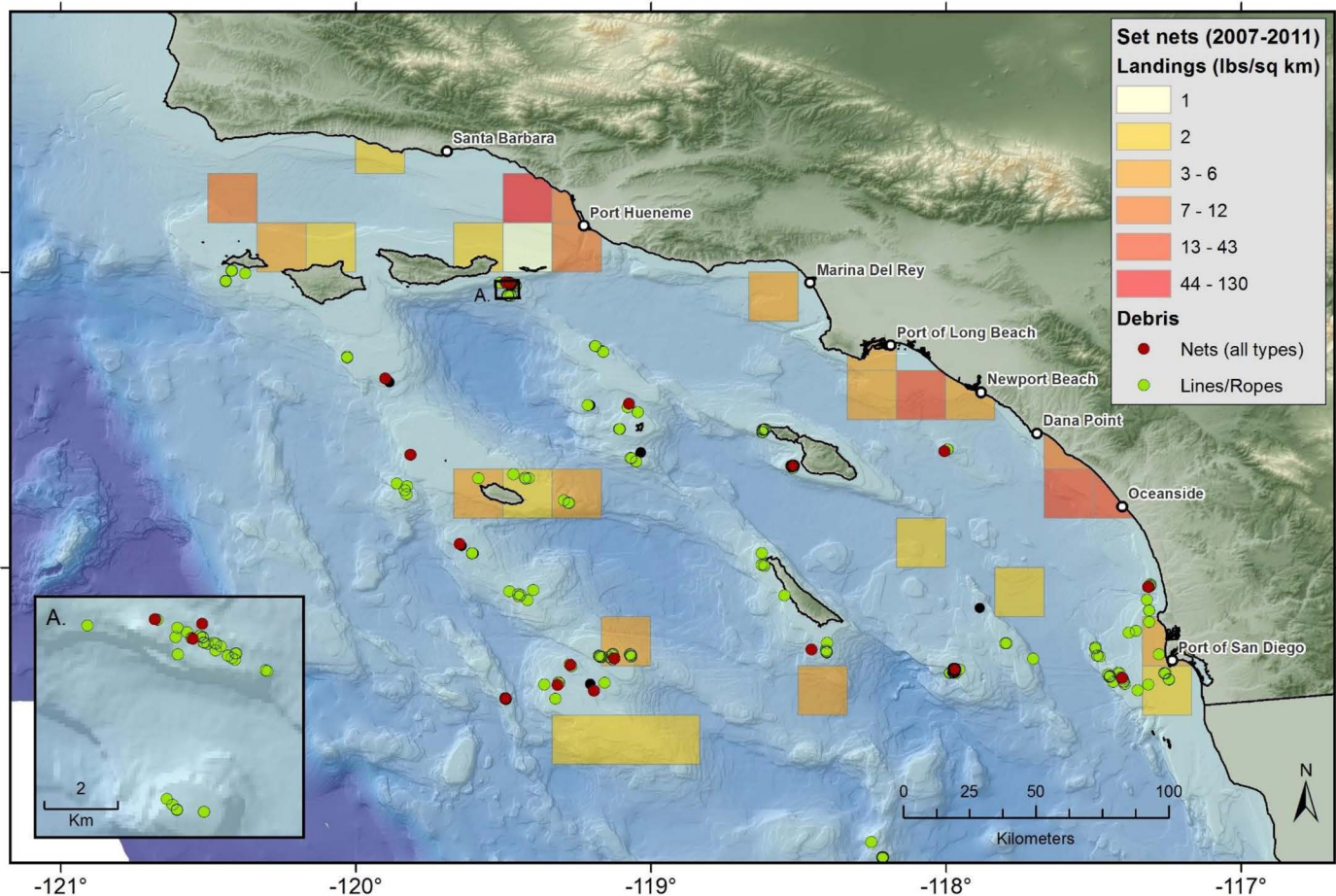

Supplement: Supplemental Information 8 — Reported landings for bottom set nets by catch block from 2007–2011 in the Southern California Bight. Observations of fishing debris (specifically nets, lines, and ropes). [file peerj-06-5697-s008.pdf]
